# *BrainGENIE*: The Brain Gene Expression and Network Imputation Engine

**DOI:** 10.1038/s41398-023-02390-w

**Published:** 2023-03-22

**Authors:** Jonathan L. Hess, Thomas P. Quinn, Chunling Zhang, Gentry C. Hearn, Samuel Chen, Neuropsychiatric Consortium for Analysis and Sharing of Transcriptomes, Natalie Jane Beveridge, Vaughan Carr, Simone de Jong, Erin Gardiner, Brian Kelly, Nishantha Kumarasinghe, Roel Ophoff, Ulrich Schall, Rodney Scott, Boryana Stamova, Paul Tooney, Sek Won Kong, Murray Cairns, Ming T. Tsuang, Stephen V. Faraone, Stephen J. Glatt

**Affiliations:** 1 Department of Psychiatry & Behavioral Sciences, Norton College of Medicine at SUNY Upstate Medical University, Syracuse, NY USA; 2 Applied Artificial Intelligence Institute (A2I2), Deakin University, Geelong, Australia; 3 Department of Neuroscience & Physiology, Norton College of Medicine at SUNY Upstate Medical University, Syracuse, NY USA; 4 Computational Health Informatics Program, Boston Children’s Hospital, Boston, MA USA; 5 Department of Pediatrics, Harvard Medical School, Boston, MA USA; 6 School of Biomedical Sciences & Pharmacy, Faculty of Health, The University of Newcastle, New South Wales, Callaghan, New South Wales Australia; 7 Hunter Medical Research Institute, Newcastle, Australia; 8 Centre for Brain & Mental Health Research, The University of Newcastle, Callaghan, Australia; 9 Center for Behavioral Genomics, Department of Psychiatry, Institute for Genomic Medicine, University of California, San Diego, La Jolla, CA USA; 10 Harvard Institute of Psychiatric Epidemiology and Genetics, Boston, MA USA; 11 Schizophrenia Research Institute, Sydney, New South Wales Australia; 12 School of Biomedical Sciences & Pharmacy, College of Health, Medicine and Wellbeing, The University of Newcastle, Callaghan, New South Wales Australia; 13 Centre for Brain & Mental Health, The University of Newcastle, Callaghan, Newcastle, Australia; 14 School of Psychiatry, University of New South Wales, Kensington, NSW Australia; 15 MRC Social, Genetic and Developmental Psychiatry Centre, King’s College London, London, UK; 16 School of Medicine & Public Health, The University of Newcastle, Callaghan, Newcastle, Australia; 17 Department of Anatomy, Faculty of Medical Sciences, University of Sri Jayawardenepura, Nugegoda, Sri Lanka; 18 Schizophrenia Research Institute, Randwick, NSW Australia; 19 Faculty of Medicine, Sir John Kotelawala Defence University, Ratmalana, Sri Lanka; 20 Center for Neurobehavioral Genetics, Semel Institute for Neuroscience and Human Behavior, University of California, Los Angeles, Los Angeles, CA USA; 21 Department of Human Genetics, University of California, Los Angeles, Los Angeles, CA USA; 22 Department of Neurology, UC Davis School of Medicine, Sacramento, CA USA

**Keywords:** Genomics, Psychiatric disorders

## Abstract

In vivo experimental analysis of human brain tissue poses substantial challenges and ethical concerns. To address this problem, we developed a computational method called the Brain Gene Expression and Network-Imputation Engine (*BrainGENIE*) that leverages peripheral-blood transcriptomes to predict brain tissue-specific gene-expression levels. Paired blood–brain transcriptomic data collected by the Genotype-Tissue Expression (GTEx) Project was used to train *BrainGENIE* models to predict gene-expression levels in ten distinct brain regions using whole-blood gene-expression profiles. The performance of *BrainGENIE* was compared to *PrediXcan*, a popular method for imputing gene expression levels from genotypes. *BrainGENIE* significantly predicted brain tissue-specific expression levels for 2947–11,816 genes (false-discovery rate-adjusted *p* < 0.05), including many transcripts that cannot be predicted significantly by a transcriptome-imputation method such as *PrediXcan*. *BrainGENIE* recapitulated measured diagnosis-related gene-expression changes in the brain for autism, bipolar disorder, and schizophrenia better than direct correlations from blood and predictions from *PrediXcan*. We developed a convenient software toolset for deploying *BrainGENIE*, and provide recommendations for how best to implement models. *BrainGENIE* complements and, in some ways, outperforms existing transcriptome-imputation tools, providing biologically meaningful predictions and opening new research avenues.

## Introduction

Brain disorders cause considerable disability worldwide [1]. Typically, in vivo molecular assessment of human disease centers on the primarily affected tissue(s) or the site of pathogenesis, but that is not possible for brain disorders unless neurosurgical intervention is required. Collecting ex vivo human brain tissue in an experimental setting for neuropsychiatric research is infeasible, given the considerable risks associated with brain biopsy. There are numerous research questions that would be answered best by studying living human brain tissue, but which therefore remain unaddressed. Transcriptome imputation offers a non-invasive alternative to brain biopsy by allowing investigators to infer tissue-specific gene expression without directly assaying gene-expression levels.

*FUSION* and *PrediXcan* are two software tools that model tissue-specific effects of expression quantitative trait loci (eQTLs) on the expression of proximal genes (*cis*-eQTLs) in order to impute transcriptome profiles. These methods have been successful in prioritizing genome-wide association study (GWAS) hits and have helped reveal putative mechanisms underlying complex disorders [2–6]. With both methods, there is a striking disparity between the number of genes imputable in the brain versus tissues outside of the central nervous system (CNS): to wit, *FUSION* imputes an average of 3158 genes in the brain (range = 1604–5855 across the 12 brain tissues (including a pair of re-sampled tissues from frontal cortex [BA9] and cerebellum) compared with 5592 in non-CNS tissues; similarly, *PrediXcan* imputes an average of 4337 genes in the brain (range = 2559–6794) compared with 6262 genes (range = 1642–10,012) outside the CNS. Furthermore, the majority of genes in the brain transcriptome are not significantly predicted by either *FUSION* or *PrediXcan*, suggesting that a large amount of variance in transcriptome profiles cannot be captured by eQTLs alone. A recent addition to the suite of genotype-based transcriptome-imputation methods is *TIGAR*, which uses a Bayesian modeling framework for predicting gene expression from eQTL data. Using a data-driven nonparametric model of *cis*-eQTL signals, *TIGAR* further increases the number of imputable genes by 57.8% compared to *PrediXcan*, but *PrediXcan* was deemed the preferred method for imputing genes that have few eQTLs influencing expression heritability [7]. A Bayesian hierarchical model called *EpiXcan* builds upon *PrediXcan* by applying epigenetic annotations to optimize the weights assigned to *cis*-eQTLs and increase the predictability of gene-expression levels [8]. *EpiXcan* increased the number of genes that can be significantly predicted by 94% compared to *PrediXcan*. Among the 2894 genes for which expression levels can be significantly predicted by both methods, *EpiXcan* showed better average prediction accuracy (mean cross-validation *R*^2^ = 0.19) compared to *PrediXcan* (mean *R*^2^ = 0.16). Despite the methodological improvements that have been made by derivatives of *PrediXcan*, a common limitation with existing *cis*-eQTL-based toolsets is that they do not allow for predictions for temporal changes in tissue-specific gene expression.

Tissue-specific and tissue-dependent gene expression help differentiate between brain and peripheral tissues, but compelling evidence also shows that brain and blood exhibit comparable transcriptome profiles [9–12]. Our group systematically reviewed relevant literature on this topic, and found that gene expression profiles in blood and brain are moderately correlated (Pearson’s *r* of 0.24–0.64), with 35–80% of genes expressed in both tissues [9]. In a later study, we found empirical evidence that ~90% of weighted gene–gene interaction networks identified in prefrontal cortex transcriptomes are preserved in peripheral blood [10]. Brain and blood also show significant overlap with respect to eQTLs [11, 12], signifying that shared genetic effects (albeit with small effect-sizes) may, in part, explain the comparability of gene expression in blood and the brain. Another advantage of capitalizing on human blood transcriptomes for brain gene expression imputation is that such data are widely available in public repositories and also can be generated de novo with relative ease and cost-effectiveness. Unlike DNA variants, transcriptome profiles in blood fluctuate over time, and they may reflect valuable information about corresponding temporal changes in the brain throughout development or over the course of an exposure or intervention.

Based on this evidence and logic, we sought to capitalize on the transcriptomic similarity between the brain and blood (and the easy accessibility of blood) to make predictions about gene expression in the brain solely based on observed expression in the periphery. Simultaneously, we aimed to develop an expression-based transcriptome-imputation method that complements existing *cis*-eQTL-based transcriptome-imputation methods. We achieved these goals with the Brain Gene-Expression and Network-Imputation Engine (*BrainGENIE*), which imputes brain tissue-specific gene-expression profiles based on gene-expression profiles assayed from peripheral blood. *BrainGENIE* is implemented in the *R* statistical environment and is distributed as freely available software (https://github.com/hessJ/BrainGENIE).

*BrainGENIE* is not the first or only cross-tissue transcriptome-imputation method, but it has unique strengths that compare quite favorably with other approaches. Tissue Expression Estimation using Blood Transcriptome (*TEEBoT*), like *BrainGENIE*, uses principal components (PCs) of peripheral blood transcriptomes to predict transcriptomes of other tissues [13]. *TEEBoT* was developed using an earlier release of GTEx (v.6) data; hence, its modeling of brain tissue-specific transcriptomes was limited to cerebellum and caudate due to sample-size restrictions. In contrast, *BrainGENIE*, which was built on the larger and more recent release of GTEx (v.8), enabling transcriptome imputation for 12 brain tissues (ten different brain regions). A second related method is *B-GEX*, which also modeled brain regional transcriptomes using an older version of GTEx that included fewer donors with paired blood and brain data [14]. Moreover, *B-GEX* utilizes individual gene transcripts from blood to predict brain gene expression, which captures less variance than PCs and limits predictive power. In short, because *BrainGENIE* was built on a better training dataset, uses PCs of whole-blood gene expression to optimize predictive power, and imputes transcriptomes for more brain tissues, it matches the strengths of competing methods and overcomes some of their limitations. Since there is no equivalent blood-based transcriptome-imputation method available that has modeled all regions of the brain like *BrainGENIE*, we benchmarked *BrainGENIE* against the most popular transcriptome-imputation method, *PrediXcan*. The two methods are conceptually similar, and there are data available from *PrediXcan* for all 12 brain tissues modeled by *BrainGENIE*, enabling direct comparisons. As such, we used the methodology of *PrediXcan* as a basis for developing *BrainGENIE* so that the results from the two methods could be directly compared. Comparing these two methods helped us to understand the differences (and points of convergence) between the use of blood-based gene-expression profiles *versus* eQTLs to impute region-specific gene expression in the brain, and illuminated the relative strengths and weaknesses of each approach. In addition to delivering a convenient software toolkit for *BrainGENIE*, we describe the application of the *BrainGENIE* method to real-world peripheral blood transcriptomic data to demonstrate the convergence of *BrainGENIE*-imputed data with disease-related gene expression changes directly measured in *postmortem* brain. Lastly, we lay the groundwork for future integrations of both blood gene expression and eQTLs to maximize the prediction of the brain transcriptome.

## Methods

### Training and evaluation of BrainGENIE

**Fig. 1:**
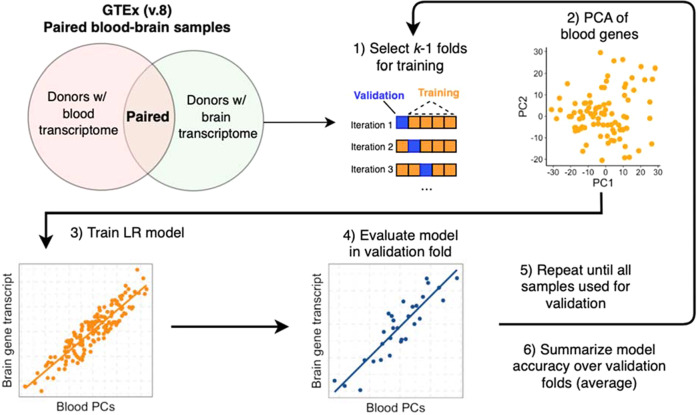
Schematic illustrating the process for training BrainGENIE using paired blood–brain transcriptome data from the GTEx dataset. BrainGENIE is trained using paired blood and brain transcriptome profiles collected by GTEx (v8) from adult donors. A single 5-fold cross-validation is performed to estimate the predictive performance of BrainGENIE separately for each brain region. BrainGENIE uses top principal components of transcriptome-wide blood-based gene expression as features to predict brain-regional gene expression levels. The metric used for prediction performance was the coefficinent of determination (*R*^2^) to measure how well predicted per-gene expression levels captured observed gene expression levels in the validation folds. Model performance was summarized over the 5 validation folds to obtain an estimate of prediction performance for BrainGENIE.

Procedures for normalizing the RNA-sequencing (RNAseq) data from GTEx are described in the Supplement (Supplementary Methods and Supplementary Fig. 1). The process used to train *BrainGENIE* is illustrated in Fig. 1. We performed a single fivefold cross-validation to estimate the predictive performance of *BrainGENIE* separately for each brain region. Paired blood–brain transcriptome profiles from GTEx donors were randomly assigned to the fivefolds. For each training *subset*, a principal component analysis (PCA) was performed on normalized blood transcriptome profiles, and linear regression was trained to predict brain tissue-specific expression levels per-gene using the top *k* = 5 (11% variance explained), *k* = 10 (41% variance explained), *k* = 20 (58% variance explained), and *k* = 40 PCs (80% variance explained) of whole-blood gene expression (resulting in fold = {1…5} by *k* = {5, 10, 20, 40} by gene = {1…*n*_genes_} linear models). The normalized transcriptome profiles in the validation subsets were projected onto the PCs of the training subsets. The linear regression model used to train *BrainGENIE* was formulated as follows: *Yi ~ β*_0_+*β*_*i*_ *X*_*i*_+*…*+ *ε*, where *Yi* represents the expression level of a gene in the brain, *β*_0_ represents the intercept, *β*_*i*_ *Xi* represents the product of the estimated regression coefficient and value of the *i*th PC, and *ε* represents the error term. Our initial work uncovered that prediction accuracies achieved by linear regression were as good as or better than elastic net regression (the model used by *PrediXcan*); linear regression is also computationally faster to train, thus was the chosen model for *BrainGENIE*. The trained models were then deployed in the validation set to estimate the predictive performance on unseen data. The metric for prediction performance was the coefficient of determination for observed and predicted *per-gene* expression levels (*R*^2^) in the hold-out fold. This process was repeated until each fold was used as the validation set, and *per-gene* prediction performance was averaged over the validation sets. In order to have a reasonable side-by-side comparison between *BrainGENIE* and *PrediXcan*, we adopted the same criterion for “significantly predicted” as adopted by *PrediXcan*; i.e., genes that could be predicted with a cross-validation (CV) *R*^2^ ≥ 0.01 and with Benjamini–Hochberg false-discovery rate-adjusted *p* value (FDR) <0.05. When comparing all models, the 40-PC *BrainGENIE* model exhibited the best performance in the training data in terms of average *R*^2^ and number of genes with significantly predicted gene expression levels, and was selected as the final model to deploy on the external test set (described below).

### Accuracy of BrainGENIE versus PrediXcan

We used two-tailed *t*-tests (alpha = 0.05) to compare the prediction accuracy of *BrainGENIE* and *PrediXcan* as indexed by Pearson’s *r* coefficients for genes that met the criterion for being “significantly predicted”. Tests were performed separately for each of the 12 brain tissues, and Benjamini–Hochberg FDR corrections were applied to resultant *p* values to adjust for multiple testing. In addition, we used Pearson’s correlation tests to assess the similarity of prediction accuracies between *BrainGENIE* and *PrediXcan* for genes that both methods significantly predicted.

### Enrichment of cross-disorder pleiotropic gene-sets

The goal of this analysis was to employ gene-set enrichment to determine if *BrainGENIE* and *PrediXcan* differed in their ability to significantly predict gene-sets that show significant association with major neuropsychiatric disorders by GWAS. Gene Ontology (GO) identifiers were obtained for 45 gene-sets identified by GWAS meta-analysis as having a shared association across eight neuropsychiatric disorders [15]. GO identifiers were annotated with HGNC gene symbols using the Molecular Signatures Database (v.6.2) [16]. We counted the number of genes in each pleiotropic gene set that were significantly predicted by either method and then performed one-tailed Fisher’s exact tests of enrichment. A significance threshold of FDR*p* < 0.05 was used to adjust for multiple comparisons.

### Concordance with neuropsychiatric-related transcriptomic signatures

To determine how well *BrainGENIE* captures brain-relevant signatures for neuropsychiatric diagnosis identified in *postmortem* brain, we performed a Pearson’s correlation analysis to determine the concordance of imputed differential-expression signatures for schizophrenia (SCZ), bipolar disorder (BD), and autism spectrum disorder (ASD) derived by *BrainGENIE* with “ground-truth” differential-expression signatures from *postmortem* brain published by the PsychENCODE Consortium and the CommonMind Consortium [17, 18]. For this analysis, we deployed *BrainGENIE* models on completely independent blood-based transcriptome datasets for SCZ [19–24] (*k* studies = 7, *n* cases = 258, *n* controls = 241), BD [24–30] (*k* studies = 8, *n* cases = 335, *n* controls = 349), and ASD [31–38] (*k* studies = 5, *n* cases = 584, *n* controls = 431). Descriptions for each dataset are provided in Supplementary Table 1. Pre-processing and normalization steps used to prepare blood-based transcriptome profiles for the SCZ, BD, and ASD datasets are described in our previously published studies [10, 39, 40]. Details of our normalization procedure are available in the supplement (Supplementary Methods). The combined set of peripheral blood transcriptome data for each disorder was then supplied to *BrainGENIE* in order to impute transcriptome profiles for the frontal cortex using the 5-, 10-, 20-, and 40-PC models.

We estimated differential gene expression (DGE) in blood between affected cases and unaffected comparison individuals *via* combined-samples mega-analyses using linear regression models that covaried for study, age, sex, and abundance of circulating leukocytes inferred using *CIBERSORT* [41]. Similarly, we estimated DGE using predicted gene-expression profiles for the frontal cortex obtained from *BrainGENIE* using the same mega-analysis approach. We applied the *PrediXcan* tool for GWAS summary statistics (*S-PrediXcan*) to obtain genetically predicted DGE effect-sizes for SCZ, BD, and ASD using the latest GWAS summary statistics for each disorder [42–44]. Transcriptome-wide DGE effect-sizes for each disorder obtained from peripheral blood mega-analyses *(t*-values)*, BrainGENIE* mega-analyses (*t*-values), and *S-PrediXcan* (*z-*scores) were then compared with DGE effect-sizes directly measured from *postmortem* brain using Pearson’s correlation test, which was chosen in order to assess the linear monotonic relationship between DGE signals derived from different methods.

## Results

### BrainGENIE prediction performance

Here, we summarize the performance of *BrainGENIE* represented by averages per brain region for the 12 brain regions being predicted. We found that *BrainGENIE* models trained with the top 40 PCs of blood-based transcriptome-wide gene expression yielded a higher average number of imputable genes per brain region relative to 5-, 10-, or 20-PC models. Thus, our summary focuses on the results derived using our 40-PC model. The prediction performance of *BrainGENIE*, measured by the average cross-validation *R*^2^, ranged from 0.03–0.56 for genes that met the criteria of significantly predicted in cross-validation (average CV *R*^2^ ≥ 0.01, FDR*p* < 0.05) (Table 1). The proportion of genes significantly predicted in the brain by *BrainGENIE* ranged from 16–59% of the total number of genes with detected expression in each brain tissue based on the GENCODE version 26 genome assembly (GRCh38) (mean number of genes = 8151; range: 2947–11,816 genes). The maximum average cross-validation prediction accuracy of *BrainGENIE* across all brain tissues ranged from *R*^2^ = 0.47–0.70. An average of 81% (range: 70–89%) of genes whose expression levels were significantly predicted by *BrainGENIE* were not significantly predicted by *PrediXcan* (Table 2). In contrast, an average of 65% of genes significantly predicted by *PrediXcan* were not significantly predicted by *BrainGENIE* (range: 50–88%) (Table 2). On average, 1672 genes were found to be significantly predicted by both *BrainGENIE* and *PrediXcan* across 12 brain tissues (range: 311 [substantia nigra]–3019 genes [cerebellum]) (Table 2). We found that expression levels of genes in the brain significantly correlated with the prediction accuracy of *BrainGENIE* (Supplementary Table 2A). Furthermore, higher expression levels of genes in the blood (Supplementary Table 2B) were significantly correlated with higher prediction accuracy of BrainGENIE, indicating that genes that are more abundant in the brain and/or blood are able to be more accurately predicted by BrainGENIE. The mean CV accuracy for *BrainGENIE* averaged across all brain regions for genes that were significantly predicted was *R*^2^ = 0.10, whereas the average CV value for *PrediXcan* was *R*^2^ = 0.15.

**Table 1:** Prediction accuracy of *BrainGENIE* (40-PC model) computed via fivefold cross-validation of the GTEx Project (v.8) release across the 12 brain tissues.

		All predicted genes					Significantly predicted genes					
Brain tissue	*n* donors (paired blood–brain transcriptomes)	# of measured genes	% of genes measured in tissue compared to all annotated genes (GRCh38)	Mean training *R*^2^	Mean CV *R*^2^	Max CV *R*^2^	*#* of genes	% of measured genes	% of genes measured in tissue compared to all annotated genes (GRCh38)	Mean training *R*^2^	Mean CV *R*^2^	Mean CV Pearson’s *r*
Amygdala	88	18,957	34	0.65	0.05	0.39	4265	22	8	0.70	0.13	0.36
Anterior cingulate cortex BA24	99	19,236	34	0.56	0.05	0.43	6799	35	12	0.59	0.11	0.33
Caudate basal ganglia	137	20,524	37	0.46	0.06	0.38	10,772	52	19	0.50	0.09	0.30
Cerebellum (Fresh frozen)	131	20,540	37	0.48	0.06	0.5	9573	47	17	0.53	0.11	0.33
Cerebellum (PAXgene preserved)	154	21,494	38	0.42	0.05	0.39	10,098	47	18	0.47	0.09	0.30
Frontal cortex (PAXgene preserved)	141	20,340	36	0.42	0.06	0.5	10,186	50	18	0.46	0.10	0.32
Frontal Cortex (Fresh frozen)	125	19,983	36	0.49	0.07	0.5	11,816	59	21	0.53	0.11	0.33
Hippocampus	122	20,189	36	0.47	0.03	0.46	4008	20	7	0.53	0.10	0.32
Hypothalamus	114	20,839	37	0.50	0.04	0.56	5749	28	10	0.56	0.11	0.33
Nucleus accumbens basal ganglia	148	20,884	37	0.43	0.05	0.46	11,252	54	20	0.47	0.09	0.30
Putamen basal ganglia	125	19,330	34	0.49	0.06	0.54	10,350	54	18	0.53	0.11	0.33
Substantia nigra	86	18,882	34	0.62	0.04	0.51	2947	16	5	0.69	0.14	0.37

The proportion of genes expressed in the whole blood accounts for 31% of all genes annotated in the GRCh38 genome assembly. The criteria for declaring genes “significantly predicted” is as follows: cross-validation [CV] *R*^2^ ≥ 0.01, CV FDR*p* ≤ 0.05. The total number of genes detected in each brain tissue for which *BrainGENIE* models were trained appears in the third column (“# of genes”). The proportion of genes that were significantly predicted by *BrainGENIE* from the total number of detected genes per brain tissue is presented in the ninth column (“% of genes”).

**Table 2:** The number of genes for which brain tissue-specific expression levels can be reliably predicted by *BrainGENIE* (40-PC model), by *PrediXcan*, or by both methods.

Brain tissue	*BrainGENIE*	*PrediXcan*	Overlap
Amygdala	4265	2787	504
Anterior cingulate cortex BA24	6799	3544	992
Caudate basal ganglia	10,772	5004	2225
Cerebellum (Fresh frozen)	9573	5753	2530
Cerebellum (PAXGene preserved)	10,098	6794	3019
Frontal cortex (PAXGene preserved)	10,186	5500	2408
Frontal Cortex (Fresh frozen)	11,816	4563	2289
Hippocampus	4008	3688	588
Hypothalamus	5749	3652	873
Nucleus accumbens basal ganglia	11,252	4851	2252
Putamen basal ganglia	10,350	4436	2067
Substantia nigra	2947	2559	311

We found overlap between brain regions in terms of the number of genes that were significantly predicted by *BrainGENIE*, which was lowest between substantia nigra and amygdala (912 genes) and highest between nucleus accumbens and frontal cortex [frozen] (7604 genes) (Supplementary Fig. 2A). The pattern of inter-regional similarity with respect to the number of genes significantly predicted by *BrainGENIE* mirrored the spatial pattern of similarity that exists between brain regions with respect to commonly expressed genes (Pearson’s *r* = 0.55, *p* = 2 × 10^−6^) (Supplementary Fig. 2B), indicating that *BrainGENIE* preserves and recapitulates the spatial relationship between areas of the brain.

The distributions of cross-validation *R*^2^ values produced by *BrainGENIE* and *PrediXcan* for all significantly predicted genes are shown in Supplementary Fig. 3. The shapes of the distributions found using *BrainGENIE* were similar to those for *PrediXcan*; however, two distinctions were consistently noted across brain tissues. First, *PrediXcan* featured heavier right-tails compared to *BrainGENIE* (Supplementary Fig. 3), indicative of *PrediXcan* having more genes with higher prediction accuracies. In contrast, *BrainGENIE* produced curves whose maxima were consistently shifted to the right relative to those of *PrediXcan*, indicative of greater average predictability with *BrainGENIE*. We found a significant association between the cross-validation performance of *BrainGENIE* in GTEx and the average RNA qualities of brain tissues (Supplementary Fig. 4A), wherein brain tissues that had better RNA quality of the brain tissues exhibited a larger number of imputable genes by *BrainGENIE* (Pearson’s *r* = 0.64, *p* = 0.024, Supplementary Fig. 4B).

Among those genes that are significantly predicted by both methods, *PrediXcan* showed significantly better overall prediction accuracy for gene expression levels in ten brain tissues (Supplementary Table 3). Prediction accuracies were statistically indistinguishable between *BrainGENIE* and *PrediXcan* for the remaining two brain tissues: the amygdala and substantia nigra (FDR*p* > 0.05, Supplementary Table 3). When evaluating the similarity of prediction accuracy among genes that are significantly predicted by both methods, *BrainGENIE* showed a small but significantly negative correlation with *PrediXcan* for genes in the amygdala, caudate, cerebellum (PAXgene preserved), frontal cortex (PAXgene preserved and fresh frozen), putamen, and nucleus accumbens (Supplementary Table 4). This finding is indicative of the methodological designs not converging to achieve consistent predictions, hence lending support to a joint imputation-modeling approach that capitalizes on blood-based gene expression and genotypes to impute brain region-specific gene expression levels.

### Cross-disorder gene sets predicted by BrainGENIE versus PrediXcan

Supplementary Fig. 5 shows that 31 of the 45 pleiotropic gene sets for eight neuropsychiatric disorders identified by the Cross-Disorder Group of the Psychiatric Genomics Consortium [15] showed significant enrichment of genes significantly predicted by *BrainGENIE*. Genes significantly predicted by *PrediXcan* showed enrichment in 11 pleiotropic gene sets, though all 11 gene sets showed more significant enrichment of genes predicted by *BrainGENIE*. The null hypothesis of these analyses was that the number of genes significantly imputable by *BrainGENIE* or by *PrediXcan* does not relate to the membership of genes to cross-disorder-associated gene-sets. The alternate hypotheses are that either (or both) algorithms allow for significant imputation of more genes that participate in cross-disorder-associated gene ontologies than expected by chance.

### Concordance of DGE changes related to neuropsychiatric disorders

**Fig. 2:**
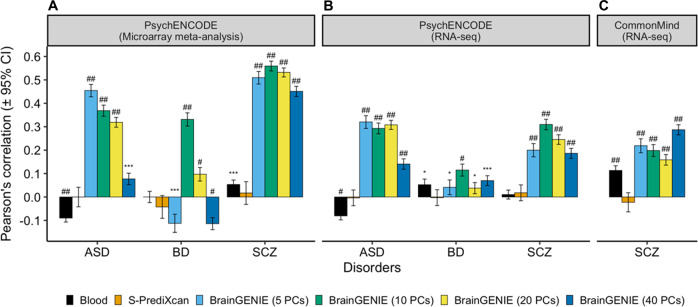
BrainGENIE recapitulates disorder-related changes in gene expression found in postmortem brain. Concordance of case-control differential gene expression (DGE) signals obtained by *BrainGENIE* and *S-PrediXcan* compared to **A** DGE signals derived from postmortem cortical microarray meta-analyses for ASD, BD, and SCZ, **B** DGE signals derived from RNA-sequencing analysis for ASD, BD, and SCZ by the PsychENCODE Consortium, and **C** DGE signals obtained from postmortem prefrontal cortex RNA-sequencing analysis for SCZ by the CommonMind Consortium. ASD autism spectrum disorder, BD bipolar disorder, and SCZ schizophrenia. Symbols for significance thresholds: *p* < 0.05 (*), FDR*p* ≤ 1 × 10^−5^ (***), FDR*p* ≤ 1 × 10^−10^ (#), FDR*p* ≤ 1 × 10^−20^ (##).

Transcriptome-wide DGE effect-sizes measured in peripheral blood for BD and SCZ demonstrated small but significant correlations with DGE effect-sizes directly measured in *postmortem* brain (Fig. 2) (Pearson’s *r* range: 0.05–0.11). The total number of genes that were represented in our analysis are provided in Supplementary Table 5. Conversely, DGE effect-sizes for ASD measured in peripheral blood showed significant inverse correlations with DGE effect-sizes directly measured in postmortem brain (Fig. 2) in the PsychENCODE microarray meta-analysis (Pearson’s *r* = −0.09, *p* = 1.7 × 10^−25^) and RNAseq analysis (Pearson’s *r* = −0.08, *p* = 3.3 × 10^−20^), which may reflect age differences between samples considering that individuals in the peripheral blood datasets were predominantly children whereas those in the *postmortem* brain studies were predominantly adults. DGE effect-sizes found using *S-PrediXcan* were not significantly correlated with postmortem brain DGE effect-sizes for SCZ, BD, or ASD (Fig. 2). Conversely, DGE effects estimated from predicted genes’ expression profiles in brain using *BrainGENIE* were significantly correlated with results directly measured in *postmortem* brain for ASD, BD, and SCZ (Fig. 2). The strongest correlation that emerged was between DGE effect-sizes obtained using *BrainGENIE* (ten PCs) and DGE effect-sizes directly measured in *postmortem* brain for SCZ found by the PsychENCODE Consortium’s microarray meta-analysis (Pearson’s *r* = 0.56, 95% CI: 0.54–0.58, *n* genes = 4130, *p* < 1.0 × 10^−300^, Fig. 2A). The DGE correlations for ASD, BD, and SCZ found between *BrainGENIE* and postmortem brain showed significant replication in an independent PsychENCODE Consortium cohort profiled via RNAseq (Fig. 2B). Furthermore, the convergence between *BrainGENIE*-imputed and measured *postmortem* brain DGE effect-sizes for SCZ was replicated in a second independent cohort from the CommonMind Consortium, with the strongest concordance found for the 40-PC model of *BrainGENIE* (Pearson’s *r* = 0.28, 95% CI: 0.26–0.31, *n* genes = 6933, *p* = 1.1 × 10^−131^, Fig. 2C). When we restricted our analysis to genes differentially expressed in postmortem brain (FDR*p* < 0.05), the DGE agreement between *BrainGENIE*-imputed brain gene expression and *postmortem* brain measured gene expression was as good or better (Supplementary Fig. 6). Scatterplots of the DGE agreement for ASD, BD, and SCZ are provided in Supplementary Fig. 7. The DGE agreement between *BrainGENIE*-imputed brain gene expression and *postmortem* brain measured gene expression was significantly stronger than between peripheral blood and postmortem brain for ASD, BD, and SCZ for one or more *BrainGENIE* models (*z*-test *p* values <0.05, Supplementary Table 6). Similarly, measured DGE signals from the postmortem brain were significantly more concordant with those predicted from *BrainGENIE* than from *S-PrediXcan* for ASD, BD, and SCZ (Supplementary Table 6). We also evaluated results published by the PsychENCODE Consortium from their approach using *FUSION*/TWAS to prioritize genes associated with ASD, BD, and SCZ in their cohort. Similar to our findings from *S-PrediXcan*, the DGE effect-sizes imputed using *FUSION*/TWAS for ASD, BD, and SCZ were not correlated with the DGE effect-sizes directly measured in postmortem brain (ASD: Pearson’s *r* = 0.003, *p* = 0.7; BD: Pearson’s *r* = 0.005, *p* = 0.6; SCZ: Pearson’s *r* = 0.013, *p* = 0.12) [45].

## Discussion

This study introduced and benchmarked a computational method called *BrainGENIE*, which predicts brain region-specific gene expression levels based on peripheral blood transcriptomes. Over the past decade, there has been rapid growth in the number of blood-based transcriptome studies aimed at identifying biomarkers for neuropsychiatric disorders. This has led to a vast amount of useful data that may hold untapped information about the brain. Much of the raw data from published blood-based transcriptome studies of neuropsychiatric disorders can be readily downloaded from public repositories (i.e., Gene Expression Omnibus [GEO] and ArrayExpress) or made available to investigators with controlled access (i.e., dbGaP, NIMHGR, and Synapse). It is, therefore, possible to further mine those stores of transcriptome data with *BrainGENIE*, thus generating novel mechanistic hypotheses about the disease and advancing our understanding of brain disorders in a way that is clearly superior to direct correlation of blood and brain measures.

*BrainGENIE* exploits PCA as an efficient method for dimensionality-reduction while capturing more variability in the blood transcriptome for prediction compared to individual gene transcripts. PCA helps to reduce the potential of overfitting; i.e., imposing a limit on the number of input features steers a model away from “learning” random noise and fails to generalize to external data. However, the risk of overfitting is not eliminated by PCA alone. The components we derived using PCA are loosely related to “eigengenes”, which are clusters of tightly co-expressed genes identified by the data-driven clustering method Weighted Gene Co-expression Network Analysis (WGCNA) [46]. A fundamental difference between PCA and WGNCA lies in the fact that genes can load onto multiple PCs as opposed to eigengenes being derived with non-overlapping sets of genes. Future work will aim to model networks of genes that are preserved across blood and the brain to improve *BrainGENIE*’s interpretability. We applied a standard classification approach of *k*-fold cross-validation to estimate the ability of *BrainGENIE* to generalize its predictions to unseen data. Further validation is warranted to determine the generalizability of our prediction models for other brain tissues and with an external dataset that is closely matched to the demographic and technical parameters observed in our training set, should one become available. In the future, modifications to *BrainGENIE* may allow for subsets of genes to be best predicted by PCs and others by individual gene transcripts, or collections of a few (or more) closely correlated transcripts. To improve the prediction performance of *BrainGENIE*, methods that can account for nonlinear mapping between blood and brain transcriptome profiles may be incorporated.

The predictive performance of *BrainGENIE* was affected by a number of factors, including the number of samples available for training and the quality of the extracted RNAs. Brain tissues that had large sample sizes (i.e., frontal cortex) showed better prediction performance than brain regions with fewer samples (i.e., amygdala and substantia nigra). In addition, imputation performance improved with the RNA quality of the brain tissues. Differences in imputation performance also were seen between pairs of re-sampled tissue collected from the frontal cortex and cerebellum. The pair of tissues collected and preserved in PAXgene fixative exhibited lower RIN values (likely due to degraded RNAs) and lower imputation performance than the pair of brain tissues that were shipped to the University of Miami Endowment Brain Bank for collection and preservation by flash-freezing. Though the differences in imputation performance of flash-frozen and chemically preserved brain tissues were small, we recommend that investigators use the *BrainGENIE* models derived from flash-frozen brain tissues. Furthermore, there were, on average, 48 fewer donors (range: 28–63) with paired blood–brain transcriptome data in GTEx (the required input for *BrainGENIE*) than donors with paired genetic and transcriptome data (the required input for *PrediXcan*). It is challenging to draw strong conclusions about differences in model performance for individual genes between *BrainGENIE* and *PrediXcan* as *PrediXcan* had more power, and yet *BrainGENIE* outperformed *PrediXcan* on a number of benchmarks. Thus, global differences between the methods could not be explained by variation in sample size alone, and even as *BrainGENIE* was limited by a smaller sample size for model-training, we found that *BrainGENIE* could impute a substantial fraction of genes that were not imputable using *PrediXcan*. This suggests that non-genetic components of gene expression ignored by *PrediXcan* models hold significant information for transcriptome imputation.

GTEx’s transcriptomic data were derived from bulk postmortem brain tissue; thus, we did not model gene expression for any specific brain-cell-type. We instead modeled the cross-tissue overlap at the level of cell mixtures in the brain and blood for *BrainGENIE*. It is possible that commonalities seen between brain and blood gene expression could be driven by a possible shared lineage between macrophages and microglia [47, 48]. Specificity of brain cell-type transcriptome imputation with BrainGENIE may be achieved with single-cell transcriptomics, but this is not feasible at the moment due to the lack of available data.

The current version of *BrainGENIE* can predict a substantial proportion of variance in expression levels for 2947 to 11,816 genes in the human brain (depending on the brain region), which accounts for about 16 to 59% of the brain transcriptome. Prior iterations of *BrainGENIE* made continual improvements in the number of significantly predicted genes, and the variance accounted for in brain tissue-specific gene-expression levels, by moving from an approach that used individual gene transcripts in blood to predict brain gene-expression levels to the current approach of using PCs of blood gene expression. This suggests that further refinement of our models will continue to improve predictions until they reach their (unknown) maximum per-gene and per brain region. As one would expect, not all or even most genes are imputable with *BrainGENIE*, but the number of new genes that can be imputed with *BrainGENIE* and not by *PrediXcan* is considerable. The amount of overlap between *BrainGENIE* and *PrediXcan* in terms of genes whose expression can be significantly predicted was relatively small. In addition, prediction accuracies were not strongly correlated between *BrainGENIE* and *PrediXcan*, indicating that the different modeling approaches achieve partially orthogonal outcomes when predicting brain transcriptomes. This suggests that there is value to integrating *BrainGENIE* and *PrediXcan* for a combined and complementary approach to transcriptome imputation wherever genotypes and blood gene-expression data are available from the same individuals. Ideally, the strengths of multiple modeling approaches, like those in *BrainGENIE*, *PrediXcan*, and others, would be combined into a unified framework (or through the integration of outputs from multiple disparate models) to deliver a holistic portrait of the landscape of the human brain transcriptome.

We suggest the following tool selection depending on the type of data available for transcriptome imputation: (1) if only transcriptome data were available from blood, use *BrainGENIE*, (2) if only GWAS data are available, use *PrediXcan* (or a derivative), (3) if blood transcriptome *and* GWAS data are available, use *BrainGENIE* and *PrediXcan* (or a derivative) to achieve best-predicted expression levels on a per*-*gene basis depending on the target brain region. For genes that are predictable by both methods, use the method that achieved better accuracy for the specific gene being imputed in the target brain region, which is often *PrediXcan*.

Our results showed that transcriptome-wide DGE effect-sizes observed directly in postmortem brain were in better agreement with DGE effect-sizes predicted using *BrainGENIE* than with DGE effect-sizes found in analyses of peripheral blood and those imputed by *PrediXcan*. This advantage of *BrainGENIE* over peripheral blood and *PrediXcan* was most striking for SCZ but was still evident for BD and ASD. The Concordance of DGE effect-sizes between *BrainGENIE* and postmortem brain varied based on the number of PCs included in the imputation models. This finding may encourage investigators to parameterize the number of PCs for *BrainGENIE* based on the model that yields the best overall prediction accuracy. However, it is important to consider which genes are included (or lost) or more significantly predicted when adjusting the number of PCs used by *BrainGENIE*, as this can be relevant for downstream analyses. For example, a study focused on the frontal-cortical expression of the SCZ risk-gene complement component 4 (*C4A*) would favor the 20-PC model (average CV *R*^2^ = 0.20, *p* = 3.8 × 10^−7^) as it yielded higher accuracy than the *BrainGENIE* models with 5-, 10-, and 40 PCs. Alternatively, an example wherein the 40-PC model yields better imputation is the frontal cortex expression of Synaptic Ras GTPase Activating Protein 1 (*SYNGAP1*), a leading risk gene for autism (CV *R*^2^ = 0.17, *p* = 5.6 × 10^−7^). Besides recapitulating disease-related effects with *BrainGENIE*, it would be valuable to know if disease status impacts the prediction performance of our models. To test for disease-related differences in prediction performance, however, would require paired blood–brain transcriptomes from the same affected individuals with characteristics that are well-matched to the distribution of our GTEx training samples; such a sample, to our knowledge, does not yet exist, highlighting a critical priority for future research.

We applied statistical corrections to remove effects of age, sex, and genetic ancestry from the gene-expression data so that those factors would not systematically bias our models. Still, it is possible that the characteristics of the GTEx sample are not fully representative of the entire population. For example, donors in the GTEx Project were predominantly of European ancestry, hence limiting the applicability of transcriptome-imputation across diverse ancestral groups. Amassing large sample sizes that encompass a broader range of characteristics (e.g., environmental exposures, genetic background, and demographics, to name a few) would allow *BrainGENIE* to make use of more biological (useful) variability that may help increase the number of significantly predicted genes and improve variance accounted for in gene expression levels of target tissues. Increasing sample ascertainment from diverse human populations, coupled with deeper phenotyping, are strategic ways to enable more effective transcriptome-imputation modeling.

In sum, *BrainGENIE* is a validated approach to investigating brain region-specific gene-expression profiles. We demonstrated that gene-expression changes associated with disease and imputed in the brain by *BrainGENIE* were in better agreement (relative to *cis-*eQTL-based predictions of gene expression by *PrediXcan* and to gene-expression changes detected in peripheral blood) with corresponding gene-expression changes detected in studies of postmortem brain. The main challenge of transcriptome-imputation is identifying a model and set of predictor variables that can efficiently and significantly predict gene-expression levels while ensuring that downstream analyses of predicted expression levels can yield biologically meaningful results. *PrediXcan* and *FUSION*, respectively, can significantly predict an average of 18 and 16% of the brain transcriptome (compared with an average of 40% by *BrainGENIE*). Those methods have been successful in identifying novel tissue-specific dysregulation of gene expression in complex disorders. A strength of *BrainGENIE* is that it can capture regulatory impacts of genetic *and* non-genetic factors on gene expression that are not yet modeled by *cis*-eQTL-based methods. *BrainGENIE* fills a void in the study of the brain transcriptome by both allowing analyses of genes that were not previously imputable and improving the predictability of disease-relevant gene sets that *PrediXcan* can only partially impute.

Though we showed that *BrainGENIE* has advantages over conceptually similar methods, our intention is for it to serve as a complement to genetic-based transcriptome-imputations methods. In practice, our recommendation would be to integrate *BrainGENIE* with other methods, whenever possible, to boost confidence in gene-disease associations, hence permitting a deeper understanding of complex phenotypes. As such, *BrainGENIE* offers an important function in systems-level research into the brain and serves as a valuable hypothesis-generating tool for mechanistic studies. Potential applications of *BrainGENIE* are far-reaching and would be best indicated (relative to *PrediXcan* and *FUSION*) to study gene expression longitudinally, including: across developmental timepoints of the brain, pre- and post-exposure (e.g., environmental risks, traumatic life experiences), and modeling the effects of medication or other clinical interventions. *BrainGENIE* also could be used to impute brain region-specific transcriptomes at any point in a person’s lifetime, opening the possibility that we could find causal and longitudinal mechanisms underlying neuropsychiatric disease. The reach of our toolset can be extended with additional developments to achieve reliable imputations of cell-type specific transcriptomes, and transcriptomes of other inaccessible tissues, as well as models of alternatively spliced mRNAs and short and long noncoding RNAs, all of which are feasible objectives.

## Supplementary information

Supplement

## Data Availability

Data and source code can be accessed from the following GitHub repository: https://github.com/hessJ/BrainGENIE.

## Code availability

Data and source code can be accessed from the following GitHub repository: https://github.com/hessJ/BrainGENIE.
