# Supplementary material for: BrainGENIE: The Brain Gene Expression and Network Imputation Engine
Source: Transl Psychiatry. 2023 Mar 22;13:98. doi: 10.1038/s41398-023-02390-w (PMC10033657; doi:10.1038/s41398-023-02390-w)
Supplement: Supplementary file 1 — Supplement [file 41398_2023_2390_MOESM1_ESM.docx]

Table of Contents

[Supplementary Methods 2](#_Toc125182288)

[*Demographics and Transcriptomic Data from the Genotype Tissue Expression Project* 2](#_Toc125182289)

[*Normalization of RNAseq Data from GTEx* 2](#_Toc125182290)

[*Statistical Deconvolution of Whole Blood Transcriptomic Data from GTEx* 3](#_Toc125182291)

[*Adjusting for Confounding Variation in the GTEx Dataset* 3](#_Toc125182292)

[Supplementary Tables 5](#_Toc125182293)

[Supplementary Table 1. Description of peripheral blood case-control datasets included in our differential gene expression mega-analyses. 5](#_Toc125182294)

[Supplementary Table 2. Correlation tests showing the relationship between gene expression levels (in brain or whole blood) and the accuracy of BrainGENIE in predicting brain-regional gene expression levels. Asterisks (*) denote correlations that were statistically significant (ps<0.05). 6](#_Toc125182295)

[Supplementary Table 3. Summary of two-tailed *t*-tests comparing the prediction accuracy for genes between BrainGENIE and PrediXcan. The analysis was restricted to genes that both methods can significantly predict. 7](#_Toc125182296)

[Supplementary Table 4. Summary statistics from Pearson’s correlation tests evaluating the similarity of prediction accuracies between *BrainGENIE* and *PrediXcan* for genes that both methods could significantly predict. 8](#_Toc125182297)

[Supplementary Table 5A. The number and percentage of genes measured in *postmortem* prefrontal cortex studies by PsychENCODE and CommonMind Consortium that were significantly predicted by *BrainGENIE*. Genes that were significantly predicted by *BrainGENIE* were included in our analysis in which we estimated the concordance of differential gene expression effect sizes between “ground truth” estimates derived from *postmortem* brain with differential gene expression effect sizes derived from *BrainGENIE* imputations. Abbreviations: autism (ASD), schizophrenia (SCZ), bipolar disorder (BD). 9](#_Toc125182298)

[Supplementary Table 5B. The number of genes available for our analysis in which we estimated the concordance of differential gene expression effect sizes between “ground truth” estimates derived from *postmortem* brain datasets with differential gene expression effect sizes derived from blood, *BrainGENIE* imputations, or *S-PrediXcan*. Abbreviations: autism (ASD), schizophrenia (SCZ), bipolar disorder (BD). 9](#_Toc125182299)

[Supplementary Table 6. Statistical test for differences in concordance of differential gene expression signals obtained from *BrainGENIE*, peripheral blood, and *S-PrediXcan* relative to independent *postmortem* brain cohorts (PsychENCODE, CommonMind). Results that remained significant after multiple testing correction appear in bold. 10](#_Toc125182300)

[Supplementary Figures 12](#_Toc125182301)

[Supplementary Figure 1 12](#_Toc125182302)

[Supplementary Figure 2 13](#_Toc125182303)

[Supplementary Figure 3. 14](#_Toc125182304)

[Supplementary Figure 4 15](#_Toc125182305)

[Supplementary Figure 5. 16](#_Toc125182306)

[Supplementary Figure 6 17](#_Toc125182307)

[Supplementary Figure 7 20](#_Toc125182308)

[Literature Cited 20](#_Toc125182309)

## Supplementary Methods

### *Demographics and Transcriptomic Data from the Genotype Tissue Expression Project*

RNA-sequencing (RNAseq) data were obtained from *postmortem* brain tissue and whole blood of 267 adult human donors. These data were generated as part of the GTEx Project (v.8), and were downloaded from the dbGaP repository (phs000424.v8.p2). Tissues selected for the GTEx Project were from donors free of brain pathology. The inclusion criteria for donors were as follows: a body mass index (BMI) of 18.5 – 35, tissue collected within 24 hours of time of death, no blood transfusions within 48 hours of time of death, no metastatic cancer, no chemotherapy or radiation within two years of time of death, and no communicable diseases that would disqualify donors from tissue donation. Transcriptome profiles were available for 12 brain tissues, two of which were collected at the same time as the donor non-brain tissue and preserved in PAXgene tissue kits (cerebellum and frontal cortex), and 10 tissues were collected and flash frozen at the University of Miami Endowment Brain Bank from whole brains that were left unfixed and shipped on wet ice (amygdala, anterior cingulate cortex, caudate, cerebellum [re-sampling], frontal cortex [re-sampling], hippocampus, hypothalamus, nucleus accumbens, putamen, and substantia nigra). Genotype-based principal components that captured genetic ancestry were available for 218 donors. Some brain tissues were not available from some donors; hence, pairs of blood-brain transcriptome profiles ranged from *n*=86 – 153. Ages of donors ranged from 20 – 70 years old with the distribution skewed toward older persons (50% over the age of 61 years, mean age=57.9 years). Approximately 30% of donors were female. Donors were recorded predominantly as of European-American ancestry (*n*=199, 91%), with 8% recorded as African American (*n*=17), and less than 1% recorded as Asian, Alaska Native, or American Indian (*n*=2).

### *Normalization of RNAseq Data from GTEx*

Total RNA was extracted from whole blood stored in PAXgene Blood RNA (Qiagen®) tubes. Total RNA was extracted from PAXgene fixed tissues sampled from frontal cortex and cerebellum at the same time as the donor non-brain tissues. Whole brains had been shipped on wet ice to the University of Miami Endowment Brain Bank where 10 brain tissues were sampled (including re-sampled frontal cortex and cerebellum as close to original site) and flash frozen. All RNA samples that were used for RNAseq had RNA integrity number (RIN) values ≥ 6.0 as measured by Agilent Bioanalyzer. Additional details related to tissue collection, library preparation, and sequencing were previously described by the GTEx Consortium (1). Gene-level read counts were summarized based on the Gencode 26 (GRCh38) transcript model. We took the following steps pre-process gene-counts for analysis, including: retain genes with > 0.1 read per kilobase per million (RPKM) and ≥5 read counts in at least 10 donors, quantile-normalize RPKM to adjust for between-sample variation (*limma* v.3.24.3) (2), and inverse-rank normalization.

### *Statistical Deconvolution of Whole Blood Transcriptomic Data from GTEx*

*CIBERSORT* (v.1.04) was used to estimate the relative abundance of circulating blood-cell subtypes across GTEx donors based on expression of cell-type-specific marker genes (3). *CIBERSORT* uses a support vector regression model to predict abundance of cell types based on prior knowledge of cell abundance and gene-expression levels in a reference panel. For this analysis, we used a reference panel referred to as “LM22”, which was comprised of expression levels for 547 genes and measured abundances of 22 human hematopoietic cells. We then performed principal components analysis (PCA) on the profiles generated by *CIBERSORT* to identify a smaller number of components that explain variation in leukocyte abundances across GTEx donors.

### *Adjusting for Confounding Variation in the GTEx Dataset*

Covariates used by the GTEx Project in their eQTL analyses (including genotyping principal components, sequencing platform, sequencing protocol, and inferred hidden factors of gene expression profiles) were downloaded from the GTEx Project website (4, 5). Linear regression was used to derive *residual gene expression values* for blood and brain by removing variance attributed to age, sex, the top three genotype-derived principal components to adjust for ancestry, PCR method used in library preparation, RNAseq platform, RIN, the top 15 factors derived from the probabilistic estimation of expression residuals (PEER). For whole blood, we included three additional covariates (defined as the top 3 PCs from *CIBERSORT*) to adjust for between-donor variation in circulating leukocyte abundance (**Supplementary Figure 1**). The residualized data, having had confounding variation partialled out, were used for building prediction models of brain gene expression.

#### Data Import and Quality Control of Array Data

We applied a standardized pre-processing pipeline for microarray data that we previously developed to convert raw probe intensities into a suitable format for meta- and mega-analysis of multiple studies (6). The following steps were applied to unprocessed microarray data sets acquired from our literature search: (1) conventional or GC-corrected robust multi-array averaging (RMA) performed on a study-wise basis to exon array and gene chips from Affymetrix, respectively (using the *R* package *affy* (7)), (2) adjusting probe-level data for background intensities based on negative control probes available on Illumina microarrays (using the *R* package *limma* (2)), (3) log_2_ transformation to stabilize variance, and (4) quantile normalization to minimize between-sample variation. Microarray probes were mapped to HGNC gene symbols. Probes that did not map to a known a HGNC gene symbol were discarded as these have unknown biological relevance. Sometimes a gene was matched to multiple probes, in which case we computed the median normalized expression, thus converting probe-level microarray data to gene-level expression values. Gene expression values were z-transformed (mean=0, s.d.=1) *per* study to minimize unwanted variation due to platform differences. Standardized gene expression data were merged across studies based on common gene symbols.

## Supplementary Tables

## **Supplementary Table 1**. Description of peripheral blood case-control datasets included in our differential gene expression mega-analyses.

| **Schizophrenia datasets** |  |  |  |  |  |
| --- | --- | --- | --- | --- | --- |
| **Study ID** | **Platform** | **# of cases** | **# of controls** | **% male** | **Mean age in years (s.d.)** |
| Gardiner et al. (2013) (8) | Illumina Human HT-12 v3 BeadChip | 78 | 78 | 51.3 | 39.5 (13.0) |
| Glatt et al. (2009) (9) | Affymetrix Human Exon 1.0 ST | 13 | 8 | 66.7 | 44.1 (7.8) |
| Glatt et al. (2011) (10) | Affymetrix HG U133 Plus 2.0 | 8 | 12 | 50 | 40.8 (11.6) |
| Kumarasinghe et al. (2013) (11) | Illumina Human HT-12 v3 BeadChip | 9 | 11 | 60 | 34.5 (13.0) |
| de Jong et al. (2012) (12) | Illumina Human Ref-8 v3.0 | 15 | 21 | 72.2 | 30.1 (11.3) |
| de Jong et al. (2012) (12) | Illumina Human HT-12 v3 BeadChip | 106 | 95 | 58.7 | 39.5 (12.5) |
| Tsuang et al. (2005) (13) | Affymetrix HG U133A/Affymetrix HG U133 Plus 2.0 | 29 | 16 | 46.7 | 35.7 (9.8) |
| ***Total*** |  | **258** | **241** |  |  |
|  |  |  |  |  |  |
| **Bipolar Disorder datasets** |  |  |  |  |  |
| **Study ID** | **Platform** | **# of cases** | **# of controls** | **% male** | **Mean age in years (s.d.)** |
| Beech et al. (2010) (14) | Illumina Human-6 v2 BeadChip | 20 | 15 | 31.4 | 34.1 (10.5) |
| Bousman et al. (2010) (15) | Affymetrix Human Exon 1.0 ST | 9 | 8 | 70.6 | 43.6 (7.2) |
| Clelland et al. (2013) (16) | Affymetrix HG U133 Plus 2.0 | 26 | 25 | 100 | 36.6 (13.0) |
| Padmos et al. (2008) (17) | Affymetrix U95v2 | 5 | 6 | 45.5 | 23.6 (9.6) |
| Savitz et al. (2013) (18) | Illumina HT-12 v4 expression BeadChip | 8 | 24 | 46.9 | 35.0 (11.1) |
| Tsuang et al. (2005) (13) | Affymetrix HG U133A/Affymetrix HG U133 Plus 2.0 | 16 | 23 | 41 | 42.4 (13.5) |
| Krebs et al. (2020) (19) | Illumina HiSeq 2500 | 240 | 238 | 44.6 | 46.9 (14.1) |
| Witt et al. (2014) (20) | Affymetrix Human Exon 1.0 ST | 11 | 10 | 100 | 48.2 (11.3) |
| ***Total*** |  | **335** | **349** |  |  |
|  |  |  |  |  |  |
| **Autism datasets** |  |  |  |  |  |
| **Study ID** | **Platform** | **# of cases** | **# of controls** | **% male** | **Mean age in years (s.d.)** |
| CHARGE (21–24) | Affymetrix HG U133 Plus 2.0 | 118 | 90 | 85.6 | 3.7 (0.76) |
| Glatt et al. (2012) (25) | Illumina HumanWG-6 v3.0 | 173 | 159 | 69.2 | 2.0 (0.78) |
| Kong et al. (2012) (26) | Affymetrix HG U133 Plus 2.0/Affymetrix Human Exon 1.0 ST | 165 | 106 | 80.4 | 8.1 (4.2) |
| Alter et al. (2011) (27) | Affymetrix HG U133 Plus 2.0 | 75 | 59 | 100 | 6.6 (2.4) |
| Kong et al. (2013) (28) | Affymetrix Human Exon 1.0 ST | 53 | 17 | 80 | 9.6 (3.8) |
| ***Total*** |  | **584** | **431** |  |  |

## **Supplementary Table 2**. Correlation tests showing the relationship between gene expression levels (in brain or whole blood) and the accuracy of BrainGENIE in predicting brain-regional gene expression levels. Asterisks (*) denote correlations that were statistically significant (ps<0.05).

| **Supplementary Table 2A.** Correlation between the prediction accuracy of *BrainGENIE* and gene expression levels in brain | | | |
| --- | --- | --- | --- |
| **Brain region** | **Pearson's r** | **t-statistics** | **p-value** |
| Amygdala | 0.07 | 4.41 | *1.08E-05 |
| Anterior cingulate cortex | 0.01 | 1.14 | 0.25 |
| Caudate | 0.12 | 12.04 | *3.77E-33 |
| Cerebellum | 0.08 | 7.78 | *7.77E-15 |
| Cerebellum (PAXGene preserved) | 0.12 | 12.27 | *2.26E-34 |
| Frontal cortex (PAXGene preserved) | 0.08 | 7.79 | *7.43E-15 |
| Frontal cortex | 0.07 | 8.03 | *1.09E-15 |
| Hippocampus | -0.01 | -0.61 | 0.54 |
| Hypothalamus | 0.09 | 7.09 | *1.51E-12 |
| Nucleus accumbens | 0.09 | 9.69 | *4.09E-22 |
| Putamen | 0.16 | 16.37 | *1.73E-59 |
| Substantia nigra | -0.03 | -1.44 | 0.15 |
|  |  |  |  |
| **Supplementary Table 2B.**Correlation between the prediction accuracy of *BrainGENIE* and gene expression levels in whole blood | | | |
| **Brain region** | **Pearson's r** | **t-statistics** | **p-value** |
| Amygdala | 0.09 | 6.14 | *9.24E-10 |
| Anterior cingulate cortex | 0.03 | 2.59 | *9.74E-03 |
| Caudate | 0.07 | 7.10 | *1.35E-12 |
| Cerebellum | 0.05 | 4.56 | *5.21E-06 |
| Cerebellum (PAXGene preserved) | 0.06 | 6.35 | *2.20E-10 |
| Frontal cortex (PAXGene preserved) | 0.17 | 17.56 | *5.28E-68 |
| Frontal cortex | 0.13 | 13.71 | *1.77E-42 |
| Hippocampus | 0.00 | 0.12 | 0.90 |
| Hypothalamus | 0.12 | 9.22 | *4.06E-20 |
| Nucleus accumbens | 0.19 | 20.30 | *4.84E-90 |
| Putamen | 0.02 | 2.18 | *0.03 |
| Substantia nigra | 0.03 | 1.37 | 0.17 |

## **Supplementary Table 3.** Summary of two-tailed *t*-tests comparing the prediction accuracy for genes between BrainGENIE and PrediXcan. The analysis was restricted to genes that both methods can significantly predict.

| **Brain tissue** | **# of genes** | ***BrainGENIE* (mean Pearson's *r*)** | ***PrediXcan* (mean Pearson's *r*)** | ***t*-statistic** | ***p-*value** | **95% CI lower** | **95% CI upper** |
| --- | --- | --- | --- | --- | --- | --- | --- |
| Amygdala | 504 | 0.36 | 0.35 | 0.97 | 0.33 | -0.007 | 0.020 |
| Anterior cingulate | 992 | 0.33 | 0.35 | -4.06 | 5.11E-05 | -0.028 | -0.010 |
| Caudate | 2,225 | 0.30 | 0.34 | -11.44 | 1.01E-29 | -0.045 | -0.032 |
| Cerebellum (Fresh frozen) | 2,530 | 0.32 | 0.38 | -16.32 | 5.08E-58 | -0.062 | -0.049 |
| Cerebellum (PAXgene preserved) | 3,019 | 0.29 | 0.37 | -23.93 | 4.32E-119 | -0.082 | -0.069 |
| Frontal cortex (PAXgene preserved) | 2,408 | 0.30 | 0.34 | -13.00 | 8.27E-38 | -0.050 | -0.037 |
| Frontal cortex (Fresh frozen) | 2,289 | 0.33 | 0.35 | -5.32 | 1.12E-07 | -0.024 | -0.011 |
| Hippocampus | 588 | 0.31 | 0.33 | -3.58 | 3.68E-04 | -0.031 | -0.009 |
| Hypothalamus | 873 | 0.32 | 0.34 | -3.69 | 2.36E-04 | -0.029 | -0.009 |
| Nucleus accumbens basal ganglia | 2,252 | 0.29 | 0.34 | -15.00 | 3.06E-49 | -0.057 | -0.044 |
| Putamen basal ganglia | 2,067 | 0.32 | 0.34 | -6.36 | 2.23E-10 | -0.029 | -0.015 |
| Substantia nigra | 311 | 0.36 | 0.36 | 0.47 | 0.64 | -0.011 | 0.018 |

## **Supplementary Table 4**. Summary statistics from Pearson’s correlation tests evaluating the similarity of prediction accuracies between *BrainGENIE* and *PrediXcan* for genes that both methods could significantly predict.

| **Brain tissue** | **# of genes** | **% of genes expressed in any given area** | **Pearson's *r*** | ***t*-statistics** | ***p*-value** | **95% CI lower** | **95% CI upper** |
| --- | --- | --- | --- | --- | --- | --- | --- |
| Amygdala | 504 | 2.2 | -0.13 | -2.87 | 4.34E-03 | -0.21 | -0.04 |
| Anterior cingulate cortex BA24 | 992 | 4.3 | -0.02 | -0.47 | 6.37E-01 | -0.08 | 0.05 |
| Caudate basal ganglia | 2,225 | 9.6 | -0.07 | -3.28 | 1.04E-03 | -0.11 | -0.03 |
| Cerebellum (Fresh frozen) | 2,530 | 10.9 | -0.03 | -1.38 | 1.69E-01 | -0.07 | 0.01 |
| Cerebellum (PAXgene preserved) | 3,019 | 13 | -0.05 | -2.76 | 5.81E-03 | -0.09 | -0.01 |
| Frontal cortex (PAXgene preserved) | 2,408 | 10.4 | -0.10 | -4.99 | 6.47E-07 | -0.14 | -0.06 |
| Frontal cortex (Fresh frozen) | 2,289 | 9.9 | -0.08 | -3.96 | 7.79E-05 | -0.12 | -0.04 |
| Hippocampus | 588 | 2.5 | -0.02 | -0.53 | 5.98E-01 | -0.10 | 0.06 |
| Hypothalamus | 873 | 3.8 | -0.02 | -0.49 | 6.27E-01 | -0.08 | 0.05 |
| Nucleus accumbens basal ganglia | 2,252 | 9.7 | -0.06 | -2.89 | 3.92E-03 | -0.10 | -0.02 |
| Putamen basal ganglia | 2,067 | 8.9 | -0.09 | -4.30 | 1.80E-05 | -0.14 | -0.05 |
| Substantia nigra | 311 | 1.3 | -0.03 | -0.51 | 6.08E-01 | -0.14 | 0.08 |

**Caption**: The percentage of genes expressed in at least one of the 48 tissues profiled in GTEx (v8) that were significantly predicted by *BrainGENIE* appear in the second column.

## **Supplementary Table 5A**. The number and percentage of genes measured in *postmortem* prefrontal cortex studies by PsychENCODE and CommonMind Consortium that were significantly predicted by *BrainGENIE*. Genes that were significantly predicted by *BrainGENIE* were included in our analysis in which we estimated the concordance of differential gene expression effect sizes between “ground truth” estimates derived from *postmortem* brain with differential gene expression effect sizes derived from *BrainGENIE* imputations. Abbreviations: autism (ASD), schizophrenia (SCZ), bipolar disorder (BD).

| **Disorder** | **Postmortem brain dataset** | **Total # of measured genes** | **# of measured genes significantly predicted by BrainGENIE** | **% of measured genes significantly predicted by BrainGENIE** | **# of genes at FDR*p*<0.05)** |
| --- | --- | --- | --- | --- | --- |
| SCZ | PsychENCODE (microarray) | 12,383 | 6,302 | 51 | 2,043 |
| ASD | PsychENCODE (microarray) | 16,182 | 7,964 | 49 | 2,471 |
| BD | PsychENCODE (microarray) | 12,377 | 6,301 | 51 | 518 |
| SCZ | PsychENCODE (RNA-seq) | 25,772 | 9,429 | 37 | 4,821 |
| ASD | PsychENCODE (RNA-seq) | 25,772 | 9,429 | 37 | 1,611 |
| BD | PsychENCODE (RNA-seq) | 25,772 | 9,429 | 37 | 1,119 |
| SCZ | CommonMind (RNA-seq) | 16,423 | 8,448 | 51 | 573 |

## **Supplementary Table 5B**. The number of genes available for our analysis in which we estimated the concordance of differential gene expression effect sizes between “ground truth” estimates derived from *postmortem* brain datasets with differential gene expression effect sizes derived from blood, *BrainGENIE* imputations, or *S-PrediXcan*. Abbreviations: autism (ASD), schizophrenia (SCZ), bipolar disorder (BD).

| **Disorder** | **Dataset** | **# of genes** |
| --- | --- | --- |
| SCZ | Blood | 12,904 |
| BD | Blood | 4,648 |
| ASD | Blood | 12,979 |
| SCZ | BrainGENIE (5 PC model) | 3,450 |
| SCZ | BrainGENIE (10 PC model) | 3,643 |
| SCZ | BrainGENIE (20 PC model) | 7,578 |
| SCZ | BrainGENIE (40 PC model) | 6,567 |
| BD | BrainGENIE (5 PC model) | 2,414 |
| BD | BrainGENIE (10 PC model) | 4,643 |
| BD | BrainGENIE (20 PC model) | 7,896 |
| BD | BrainGENIE (40 PC model) | 8,506 |
| ASD | BrainGENIE (5 PC model) | 3,187 |
| ASD | BrainGENIE (10 PC model) | 5,447 |
| ASD | BrainGENIE (20 PC model) | 7,183 |
| ASD | BrainGENIE (40 PC model) | 8,269 |
| SCZ | S-PrediXcan | 2,296 |
| ASD | S-PrediXcan | 3,597 |
| BD | S-PrediXcan | 3,576 |

## **Supplementary Table 6**. Statistical test for differences in concordance of differential gene expression signals obtained from *BrainGENIE*, peripheral blood, and *S-PrediXcan* relative to independent *postmortem* brain cohorts (PsychENCODE, CommonMind). Results that remained significant after multiple testing correction appear in bold.

| **PsychENCODE Consortium (Microarray meta-analysis)** | | | | | | |
| --- | --- | --- | --- | --- | --- | --- |
| **Disorder** | **Method 1** | **Method 2** | **Difference in Pearson's *r*** | ***z*-statistic** | ***p*-value** | **FDRp** |
| ASD | Blood | BrainGENIE (5 PCs) | -0.54 | -27.43 | 5.43E-166 | **1.43E-164** |
| ASD | Blood | BrainGENIE (10 PCs) | -0.47 | -29.36 | 9.96E-190 | **3.49E-188** |
| ASD | Blood | BrainGENIE (20 PCs) | -0.37 | -24.45 | 2.36E-132 | **3.55E-131** |
| ASD | Blood | BrainGENIE (40 PCs) | -0.16 | -10.72 | 3.91E-27 | **1.05E-26** |
| ASD | Blood | S-PrediXcan | -0.09 | -3.92 | 4.45E-05 | **6.77E-05** |
| ASD | BrainGENIE (5 PCs) | BrainGENIE (10 PCs) | 0.07 | 3.68 | 1.16E-04 | **1.69E-04** |
| ASD | BrainGENIE (5 PCs) | BrainGENIE (20 PCs) | 0.17 | 8.44 | 1.58E-17 | **3.70E-17** |
| ASD | BrainGENIE (5 PCs) | BrainGENIE (40 PCs) | 0.38 | 18.33 | 2.50E-75 | **1.75E-74** |
| ASD | BrainGENIE (5 PCs) | S-PrediXcan | 0.45 | 16.97 | 6.78E-65 | **3.74E-64** |
| ASD | BrainGENIE (10 PCs) | BrainGENIE (20 PCs) | 0.10 | 5.58 | 1.19E-08 | **2.16E-08** |
| ASD | BrainGENIE (10 PCs) | BrainGENIE (40 PCs) | 0.31 | 17.52 | 4.62E-69 | **2.70E-68** |
| ASD | BrainGENIE (10 PCs) | S-PrediXcan | 0.38 | 15.55 | 7.75E-55 | **3.54E-54** |
| ASD | BrainGENIE (20 PCs) | BrainGENIE (40 PCs) | 0.21 | 12.41 | 1.19E-35 | **4.02E-35** |
| ASD | BrainGENIE (20 PCs) | S-PrediXcan | 0.28 | 11.67 | 9.40E-32 | **2.99E-31** |
| ASD | BrainGENIE (40 PCs) | S-PrediXcan | 0.07 | 2.86 | 2.11E-03 | **2.71E-03** |
| BD | Blood | BrainGENIE (5 PCs) | 0.11 | 4.81 | 7.43E-07 | **1.20E-06** |
| BD | Blood | BrainGENIE (10 PCs) | -0.33 | -16.86 | 4.48E-64 | **2.35E-63** |
| BD | Blood | BrainGENIE (20 PCs) | -0.10 | -5.05 | 2.24E-07 | **3.73E-07** |
| BD | Blood | BrainGENIE (40 PCs) | 0.11 | 6.34 | 1.12E-10 | **2.17E-10** |
| BD | Blood | S-PrediXcan | 0.04 | 1.54 | 6.19E-02 | 6.98E-02 |
| BD | BrainGENIE (5 PCs) | BrainGENIE (10 PCs) | -0.44 | -17.68 | 2.83E-70 | **1.86E-69** |
| BD | BrainGENIE (5 PCs) | BrainGENIE (20 PCs) | -0.21 | -8.43 | 1.78E-17 | **4.07E-17** |
| BD | BrainGENIE (5 PCs) | BrainGENIE (40 PCs) | 0.00 | 0.06 | 4.78E-01 | **4.78E-01** |
| BD | BrainGENIE (5 PCs) | S-PrediXcan | -0.07 | -2.21 | 1.36E-02 | **1.63E-02** |
| BD | BrainGENIE (10 PCs) | BrainGENIE (20 PCs) | 0.23 | 11.18 | 2.63E-29 | **7.66E-29** |
| BD | BrainGENIE (10 PCs) | BrainGENIE (40 PCs) | 0.45 | 21.82 | 7.86E-106 | **7.50E-105** |
| BD | BrainGENIE (10 PCs) | S-PrediXcan | 0.37 | 13.01 | 5.26E-39 | **1.84E-38** |
| BD | BrainGENIE (20 PCs) | BrainGENIE (40 PCs) | 0.21 | 10.63 | 1.06E-26 | **2.77E-26** |
| BD | BrainGENIE (20 PCs) | S-PrediXcan | 0.14 | 4.83 | 6.75E-07 | **1.11E-06** |
| BD | BrainGENIE (40 PCs) | S-PrediXcan | -0.07 | -2.55 | 5.37E-03 | **6.55E-03** |
| SCZ | Blood | BrainGENIE (5 PCs) | -0.46 | -24.43 | 4.24E-132 | **5.57E-131** |
| SCZ | Blood | BrainGENIE (10 PCs) | -0.51 | -31.62 | 8.66E-220 | **4.55E-218** |
| SCZ | Blood | BrainGENIE (20 PCs) | -0.48 | -32.34 | 8.90E-230 | **9.35E-228** |
| SCZ | Blood | BrainGENIE (40 PCs) | -0.40 | -25.63 | 3.88E-145 | **8.14E-144** |
| SCZ | Blood | S-PrediXcan | 0.04 | 1.38 | 8.41E-02 | **9.29E-02** |
| SCZ | BrainGENIE (5 PCs) | BrainGENIE (10 PCs) | -0.05 | -2.89 | 1.95E-03 | **2.52E-03** |
| SCZ | BrainGENIE (5 PCs) | BrainGENIE (20 PCs) | -0.02 | -1.34 | 8.97E-02 | 9.82E-02 |
| SCZ | BrainGENIE (5 PCs) | BrainGENIE (40 PCs) | 0.06 | 3.30 | 4.89E-04 | **6.66E-04** |
| SCZ | BrainGENIE (5 PCs) | S-PrediXcan | 0.49 | 17.62 | 8.56E-70 | **5.29E-69** |
| SCZ | BrainGENIE (10 PCs) | BrainGENIE (20 PCs) | 0.03 | 1.88 | 3.00E-02 | **3.47E-02** |
| SCZ | BrainGENIE (10 PCs) | BrainGENIE (40 PCs) | 0.11 | 7.00 | 1.30E-12 | **2.63E-12** |
| SCZ | BrainGENIE (10 PCs) | S-PrediXcan | 0.54 | 20.99 | 4.20E-98 | **3.67E-97** |
| SCZ | BrainGENIE (20 PCs) | BrainGENIE (40 PCs) | 0.08 | 5.50 | 1.90E-08 | **3.38E-08** |
| SCZ | BrainGENIE (20 PCs) | S-PrediXcan | 0.52 | 20.34 | 2.94E-92 | **2.38E-91** |
| SCZ | BrainGENIE (40 PCs) | S-PrediXcan | 0.43 | 16.49 | 2.02E-61 | **1.01E-60** |
| **PsychENCODE Consortium (RNAseq analysis)** | | | | | | |
| **Disorder** | **Method 1** | **Method 2** | **Difference in Pearson's *r*** | ***z*-statistic** | ***p*-value** | **FDRp** |
| ASD | Blood | BrainGENIE (5 PCs) | -0.43 | -22.65 | 7.62E-114 | **8.00E-113** |
| ASD | Blood | BrainGENIE (10 PCs) | -0.37 | -24.06 | 3.45E-128 | **4.02E-127** |
| ASD | Blood | BrainGENIE (20 PCs) | -0.35 | -24.48 | 1.09E-132 | **1.91E-131** |
| ASD | Blood | BrainGENIE (40 PCs) | -0.21 | -15.01 | 3.12E-51 | **1.37E-50** |
| ASD | Blood | S-PrediXcan | -0.08 | -4.01 | 3.09E-05 | **4.77E-05** |
| ASD | BrainGENIE (5 PCs) | BrainGENIE (10 PCs) | 0.06 | 2.96 | 1.54E-03 | **2.05E-03** |
| ASD | BrainGENIE (5 PCs) | BrainGENIE (20 PCs) | 0.07 | 3.79 | 7.40E-05 | **1.09E-04** |
| ASD | BrainGENIE (5 PCs) | BrainGENIE (40 PCs) | 0.21 | 11.01 | 1.80E-28 | **5.12E-28** |
| ASD | BrainGENIE (5 PCs) | S-PrediXcan | 0.35 | 14.89 | 1.93E-50 | **8.09E-50** |
| ASD | BrainGENIE (10 PCs) | BrainGENIE (20 PCs) | 0.01 | 0.89 | 1.87E-01 | **1.98E-01** |
| ASD | BrainGENIE (10 PCs) | BrainGENIE (40 PCs) | 0.16 | 9.53 | 7.56E-22 | **1.93E-21** |
| ASD | BrainGENIE (10 PCs) | S-PrediXcan | 0.29 | 13.92 | 2.29E-44 | **8.89E-44** |
| ASD | BrainGENIE (20 PCs) | BrainGENIE (40 PCs) | 0.14 | 9.06 | 6.56E-20 | **1.60E-19** |
| ASD | BrainGENIE (20 PCs) | S-PrediXcan | 0.28 | 13.61 | 1.75E-42 | **6.55E-42** |
| ASD | BrainGENIE (40 PCs) | S-PrediXcan | 0.14 | 6.65 | 1.47E-11 | **2.91E-11** |
| BD | Blood | BrainGENIE (5 PCs) | 0.01 | 0.54 | 2.95E-01 | 3.04E-01 |
| BD | Blood | BrainGENIE (10 PCs) | -0.06 | -3.47 | 2.60E-04 | **3.59E-04** |
| BD | Blood | BrainGENIE (20 PCs) | 0.01 | 0.83 | 2.02E-01 | 2.12E-01 |
| BD | Blood | BrainGENIE (40 PCs) | -0.02 | -1.07 | 1.43E-01 | 1.55E-01 |
| BD | Blood | S-PrediXcan | 0.06 | 2.59 | 4.75E-03 | **5.94E-03** |
| BD | BrainGENIE (5 PCs) | BrainGENIE (10 PCs) | -0.07 | -3.55 | 1.95E-04 | **2.73E-04** |
| BD | BrainGENIE (5 PCs) | BrainGENIE (20 PCs) | 0.00 | 0.18 | 4.31E-01 | 4.35E-01 |
| BD | BrainGENIE (5 PCs) | BrainGENIE (40 PCs) | -0.03 | -1.47 | 7.10E-02 | 7.93E-02 |
| BD | BrainGENIE (5 PCs) | S-PrediXcan | 0.04 | 1.87 | 3.08E-02 | **3.51E-02** |
| BD | BrainGENIE (10 PCs) | BrainGENIE (20 PCs) | 0.08 | 4.32 | 7.88E-06 | **1.25E-05** |
| BD | BrainGENIE (10 PCs) | BrainGENIE (40 PCs) | 0.05 | 2.66 | 3.89E-03 | **4.92E-03** |
| BD | BrainGENIE (10 PCs) | S-PrediXcan | 0.12 | 5.44 | 2.64E-08 | **4.62E-08** |
| BD | BrainGENIE (20 PCs) | BrainGENIE (40 PCs) | -0.03 | -1.97 | 2.43E-02 | **2.87E-02** |
| BD | BrainGENIE (20 PCs) | S-PrediXcan | 0.04 | 1.93 | 2.70E-02 | **3.15E-02** |
| BD | BrainGENIE (40 PCs) | S-PrediXcan | 0.07 | 3.57 | 1.76E-04 | **2.49E-04** |
| SCZ | Blood | BrainGENIE (5 PCs) | -0.19 | -10.76 | 2.74E-27 | **7.56E-27** |
| SCZ | Blood | BrainGENIE (10 PCs) | -0.30 | -19.41 | 2.97E-84 | **2.22E-83** |
| SCZ | Blood | BrainGENIE (20 PCs) | -0.24 | -16.24 | 1.28E-59 | **6.10E-59** |
| SCZ | Blood | BrainGENIE (40 PCs) | -0.18 | -11.95 | 3.29E-33 | **1.08E-32** |
| SCZ | Blood | S-PrediXcan | -0.01 | -0.38 | 3.50E-01 | 3.57E-01 |
| SCZ | BrainGENIE (5 PCs) | BrainGENIE (10 PCs) | -0.11 | -6.00 | 1.01E-09 | **1.93E-09** |
| SCZ | BrainGENIE (5 PCs) | BrainGENIE (20 PCs) | -0.05 | -2.56 | 5.19E-03 | **6.41E-03** |
| SCZ | BrainGENIE (5 PCs) | BrainGENIE (40 PCs) | 0.01 | 0.74 | 2.30E-01 | 2.39E-01 |
| SCZ | BrainGENIE (5 PCs) | S-PrediXcan | 0.18 | 8.09 | 2.99E-16 | **6.54E-16** |
| SCZ | BrainGENIE (10 PCs) | BrainGENIE (20 PCs) | 0.06 | 4.14 | 1.73E-05 | **2.72E-05** |
| SCZ | BrainGENIE (10 PCs) | BrainGENIE (40 PCs) | 0.12 | 7.75 | 4.73E-15 | **9.94E-15** |
| SCZ | BrainGENIE (10 PCs) | S-PrediXcan | 0.29 | 14.15 | 9.51E-46 | **3.84E-45** |
| SCZ | BrainGENIE (20 PCs) | BrainGENIE (40 PCs) | 0.06 | 3.89 | 5.02E-05 | **7.53E-05** |
| SCZ | BrainGENIE (20 PCs) | S-PrediXcan | 0.23 | 11.34 | 4.02E-30 | **1.21E-29** |
| SCZ | BrainGENIE (40 PCs) | S-PrediXcan | 0.17 | 8.29 | 5.72E-17 | **1.28E-16** |
| **CommonMind Consortium (RNAseq analysis)** | | | | | | |
| **Disorder** | **Method 1** | **Method 2** | **Difference in Pearson's *r*** | ***z*-statistic** | ***p*-value** | **FDRp** |
| SCZ | Blood | S-PrediXcan | 0.14 | 5.87 | 2.16E-09 | **4.05E-09** |
| SCZ | Blood | BrainGENIE (5 PCs) | -0.11 | -5.71 | 5.52E-09 | **1.02E-08** |
| SCZ | Blood | BrainGENIE (10 PCs) | -0.09 | -5.14 | 1.40E-07 | **2.37E-07** |
| SCZ | Blood | BrainGENIE (20 PCs) | -0.05 | -2.94 | 1.62E-03 | **2.12E-03** |
| SCZ | Blood | BrainGENIE (40 PCs) | -0.17 | -11.47 | 9.11E-31 | **2.81E-30** |
| SCZ | S-PrediXcan | BrainGENIE (5 PCs) | -0.24 | -9.33 | 5.34E-21 | **1.33E-20** |
| SCZ | S-PrediXcan | BrainGENIE (10 PCs) | -0.22 | -9.01 | 1.07E-19 | **2.56E-19** |
| SCZ | S-PrediXcan | BrainGENIE (20 PCs) | -0.18 | -7.62 | 1.26E-14 | **2.59E-14** |
| SCZ | S-PrediXcan | BrainGENIE (40 PCs) | -0.31 | -13.21 | 3.90E-40 | **1.41E-39** |
| SCZ | BrainGENIE (5 PCs) | BrainGENIE (10 PCs) | 0.02 | 1.03 | 1.52E-01 | 1.63E-01 |
| SCZ | BrainGENIE (5 PCs) | BrainGENIE (20 PCs) | 0.06 | 3.15 | 8.27E-04 | **1.11E-03** |
| SCZ | BrainGENIE (5 PCs) | BrainGENIE (40 PCs) | -0.07 | -3.64 | 1.37E-04 | **1.98E-04** |
| SCZ | BrainGENIE (10 PCs) | BrainGENIE (20 PCs) | 0.04 | 2.29 | 1.11E-02 | **1.33E-02** |
| SCZ | BrainGENIE (10 PCs) | BrainGENIE (40 PCs) | -0.09 | -5.22 | 8.86E-08 | **1.52E-07** |
| SCZ | BrainGENIE (20 PCs) | BrainGENIE (40 PCs) | -0.13 | -8.03 | 4.70E-16 | **1.01E-15** |

## **Supplementary Figures**


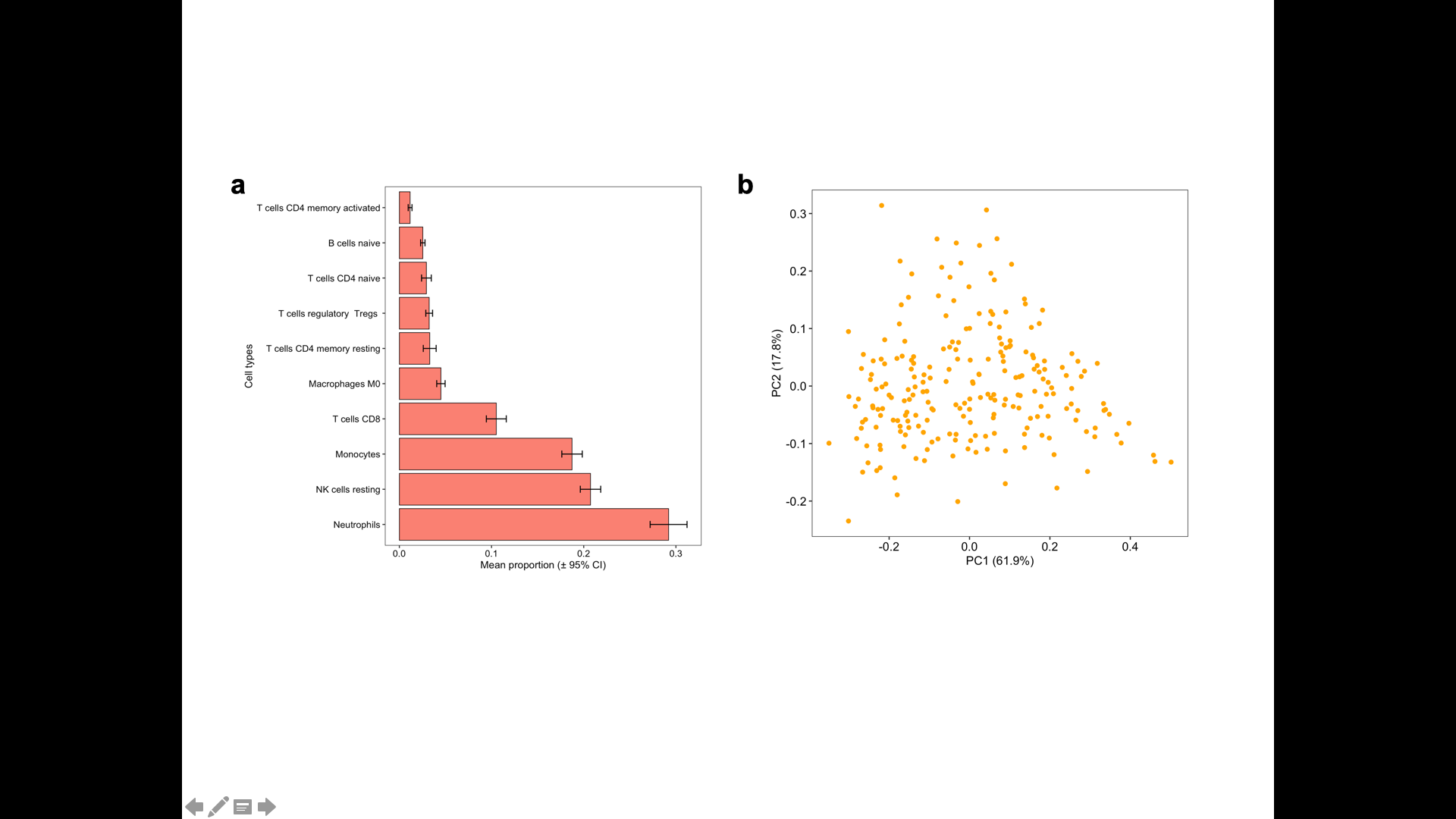


**Supplementary Figure 1.** Cell deconvolution analysis with *CIBERSORT* reveals the estimated abundance of circulating leukocytes in blood based on expression levels of cell-type marker genes assayed in whole blood from 225 donors in GTEx (v.8). (a) The estimated proportions of 10 circulating leukocytes are shown, which were averaged over 225 adult donors. The plot was limited to cell types with an average abundance ≥ 1%. (b) A scatterplot of donor scores for the first two components derived from principal components analysis (PCA) of estimated abundances of 22 leukocytes outputted from *CIBERSORT*. These component scores explained ~80% of the variance in leukocyte abundances between donors in GTEx. Donor scores for the top three principal components of blood cell abundances were regressed out of the whole blood gene expression data collected from the GTEx donors to adjust for unwanted variation in blood cell abundances that may have obfuscated (or spuriously created) cross-tissue gene expression correlations between blood and brain.

| **A** | **B** |
| --- | --- |
| 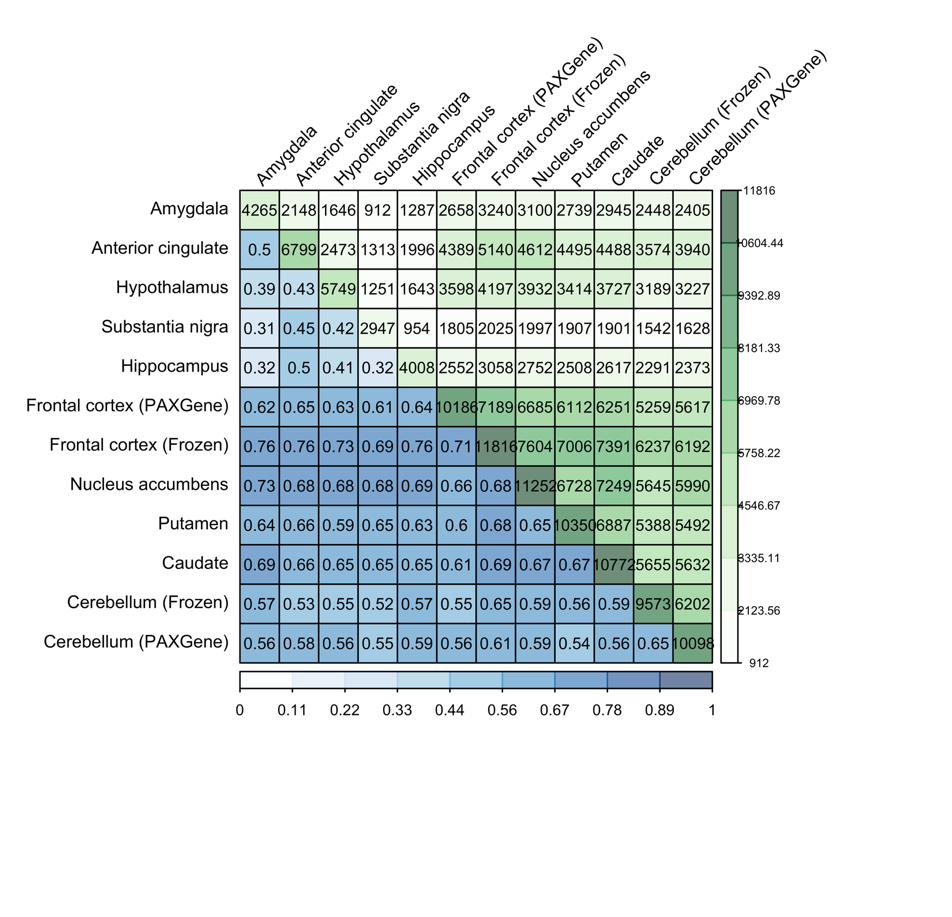 | 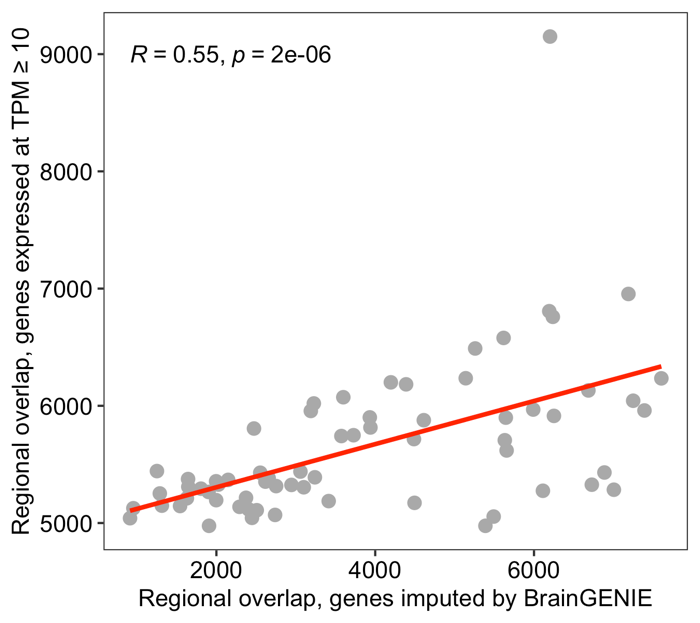 |

**Supplementary Figure 2**. *BrainGENIE* captures spatial relationships between brain tissues in GTEx. (**A**) A heatmap shows the amount of overlap between brain regions in terms of the number of genes that were significantly predicted by *BrainGENIE*. Numbers along the diagonal (upper left corner to bottom right corner) indicate the number of genes significantly predicted in each of the 12 brain tissues by BrainGENIE. Values below the diagonal (bottom left triangle, shades of blue) represent the proportion of genes from one brain tissue (the tissue with fewer significantly predicted genes) that were significantly predicted in another brain tissue (the tissue with more significantly predicted genes). Values above the diagonal (upper right triangle, shades of green) are the number of significantly predicted genes in common between pairs of brain tissues. (**B**) A scatter plot showing, on the vertical axis, the similarity between brain tissues based on their overlap in terms of expressed genes (expression threshold: ≥ 10 transcripts per million [TPM]). Plotted on the horizontal axis is the number of genes significantly predicted by BrainGENIE that were shared between brain tissues. This plot demonstrates that the expected similarity between brain tissues was partially recapitulated by *BrainGENIE* per the best-fit regression line (Pearson’s *r* =0.55, *p*<2x10^-6^).


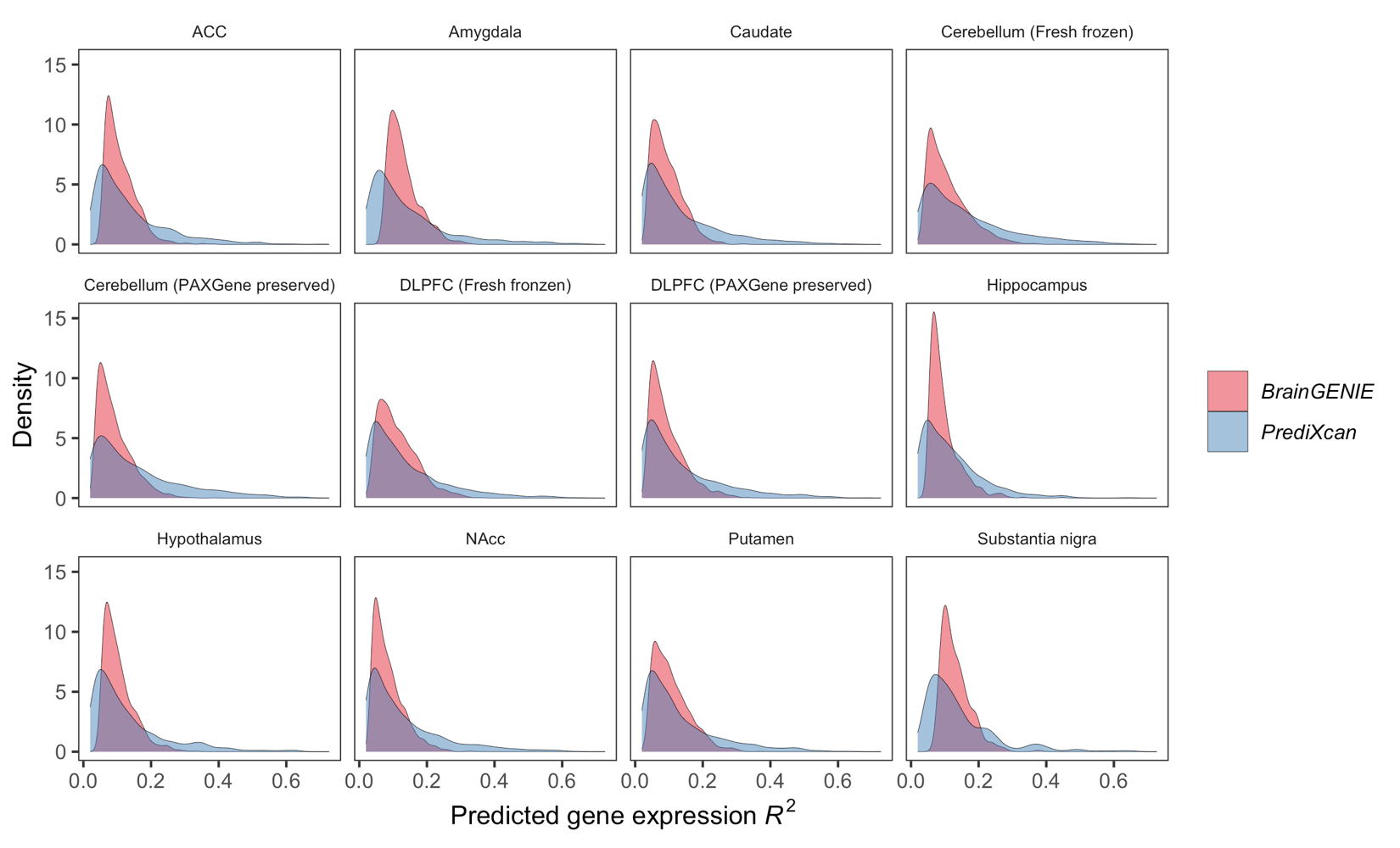


**Supplementary Figure 3**. Smoothed density kernel plots showing the distribution of model prediction performances for all genes (measured by cross-validation *R*^2^) whose expression levels in the brain were significantly predicted by *BrainGENIE* or *PrediXcan*. The shape of the density plots for *BrainGENIE* show that more genes were significantly predicted by *BrainGENIE* compared to *PrediXcan*, indicated by the heights of the light red-colored curves. The blue-colored curves, representing the prediction accuracy of *PrediXcan*, have taller and longer right tails indicating that more genes were predicted with higher accuracy compared to *BrainGENIE*.


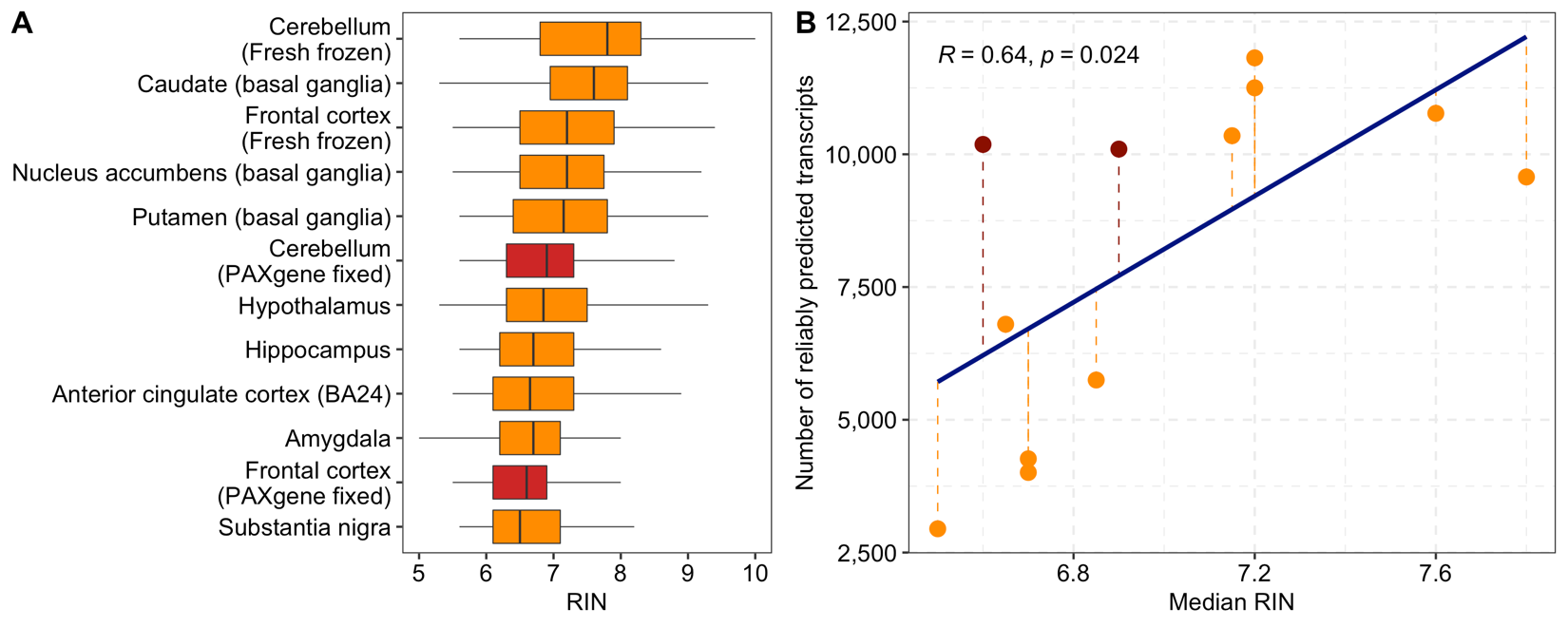


**Supplementary Figure 4**. Influence of RNA quality, measured by RNA integrity number (RIN), on the imputation performance of *BrainGENIE*. (**A**) The distribution of RIN is shown per brain tissue from GTEx (v8). Adjacently sampled brain tissues that were preserved in PAXgene tissue kits were highlighted in red; all other brain tissues were collected and fresh-frozen at the University of Miami Endowment Brain Bank. The center line in each box denotes the median, the box limits are the upper and lower quartiles, and whiskers are the interquartile range x1.5. (**B**) The relationship between median RIN and number of genes with significantly predicted expression levels was plotted. Brain tissue samples preserved in PAXgene tissue kits were highlighted in red.

**
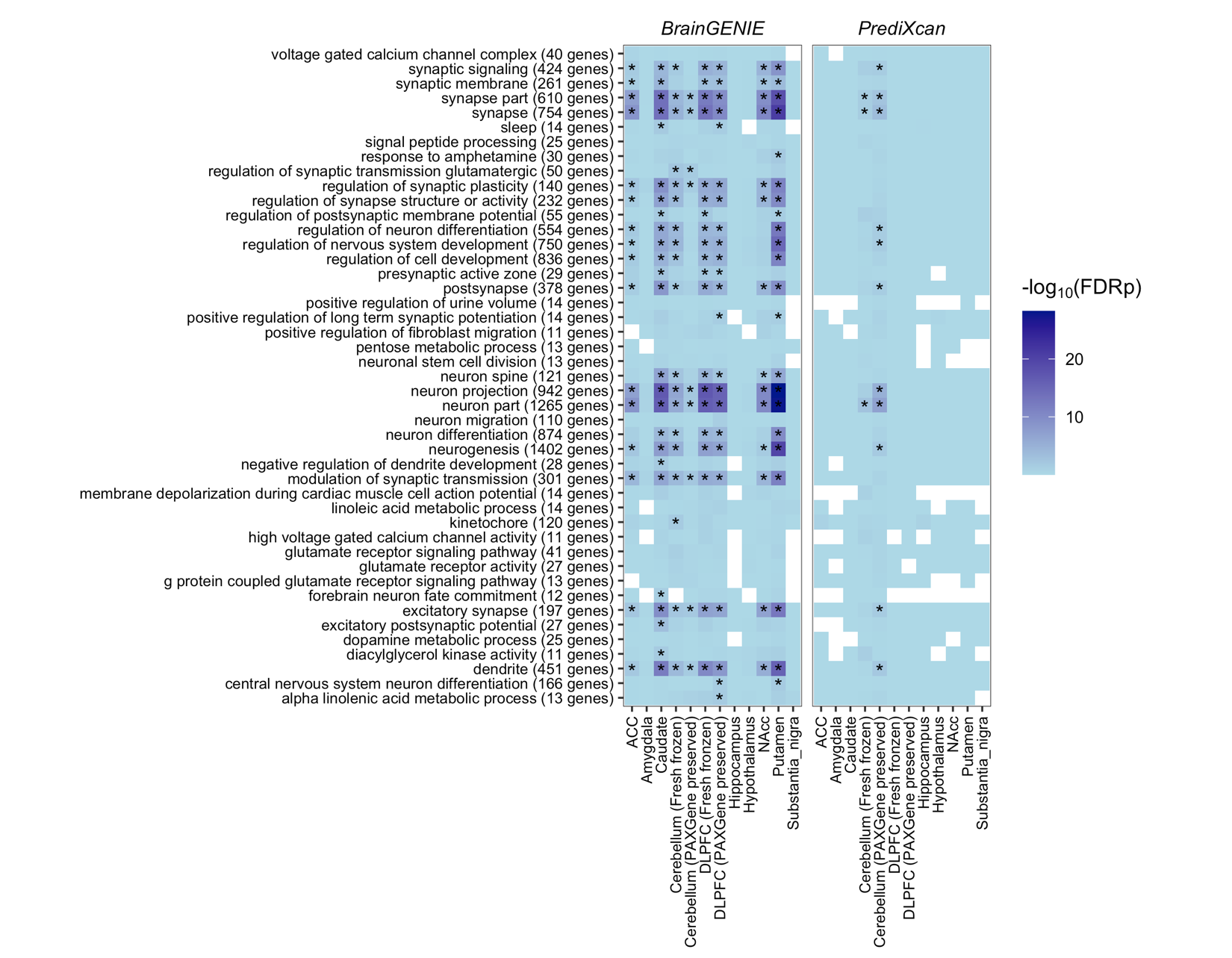
**

**Supplementary Figure 5**. A heatmap of 45 Gene Ontology (GO) gene sets known to be enriched with cross-disorder risk genes for eight neuropsychiatric disorders.(29) We performed a gene set enrichment analysis *via* one-tailed Fisher’s exact test to determine whether genes that are significantly predicted by *BrainGENIE* or *PrediXcan* are significantly over-represented in the 45 pleiotropic gene sets. Tiles that are shaded darker blue showed more significant enrichment of significantly predicted genes. Asterisks denote gene sets that were significantly over-represented by significantly predicted genes at a false-discovery rate-adjusted (FDR) *p*<0.05.


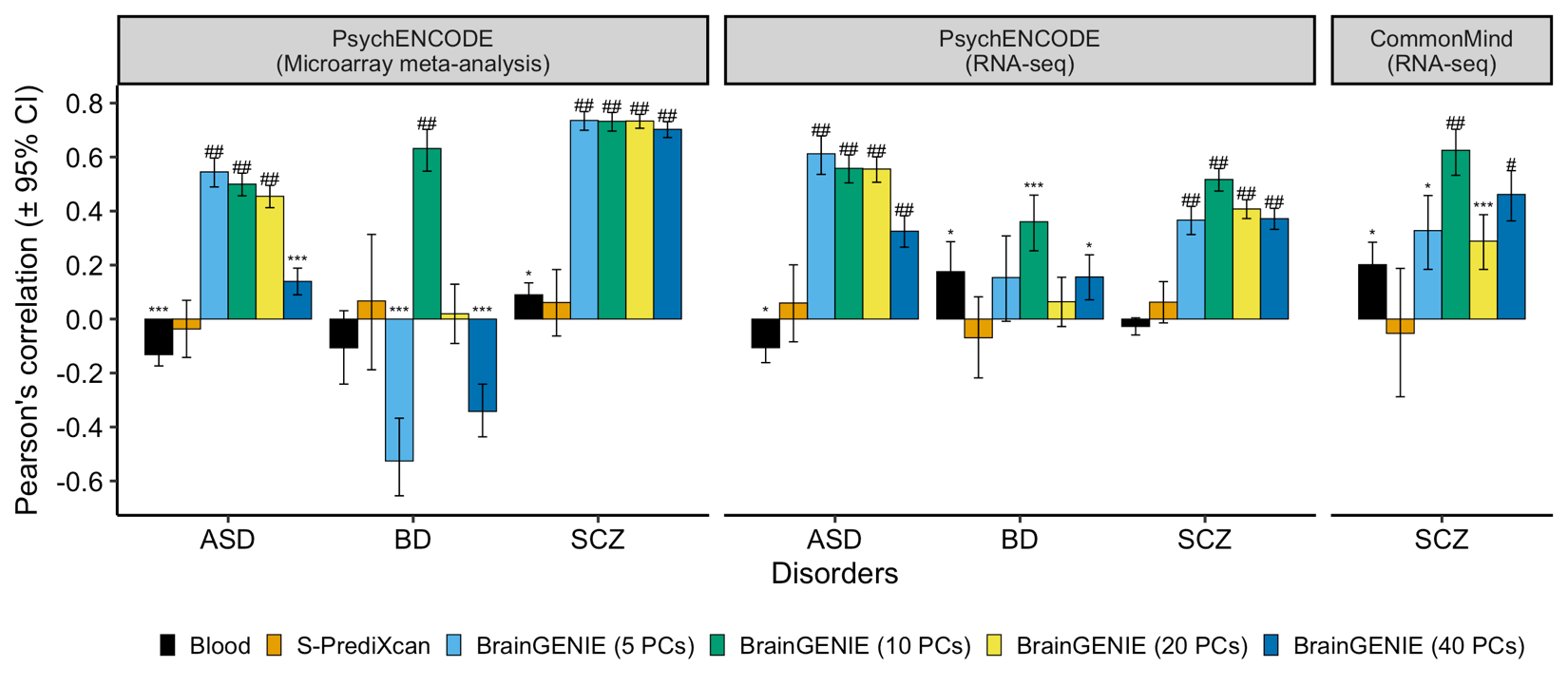


**Supplementary Figure 6**. Concordance of case-control differential gene expression (DGE) signals obtained by *BrainGENIE* and *S-PrediXcan* compared to (**A**) significant DGE signals derived from *postmortem* cortical microarray meta-analyses for ASD, BD, and SCZ at a Benjamini-Hochberg false-discovery rate (FDR) adjusted *p*<0.05, (**B**) the most significant DGE signals derived from RNA-sequencing analysis for ASD, BD, and SCZ by the PsychENCODE Consortium at a FDR*p*<0.05, and (**C**) significant DGE signals identified in *postmortem* prefrontal cortex for SCZ at a FDRp<0.05 by the CommonMind Consortium. Abbreviations: autism spectrum disorder (ASD), bipolar disorder (BD), and schizophrenia (SCZ). Symbols for significance thresholds: *p*<0.05 (*), FDR*p*≤1×10^-5^ (***), FDR*p*≤1×10^-10^ (#), FDR*p*≤1×10^-20^ (##).

| **Blood DGE vs. PsychENCODE (microarray)**  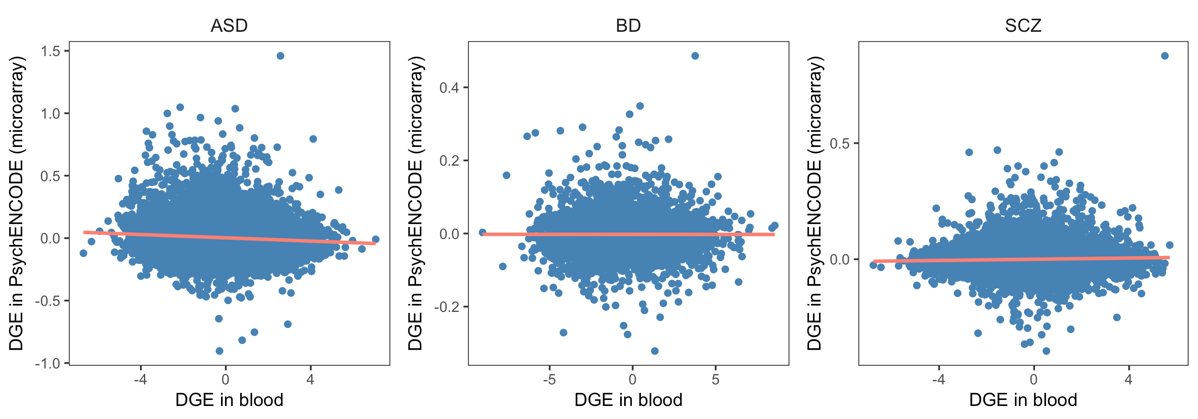 |
| --- |
| **Blood DGE vs. PsychENCODE (RNA-seq)**  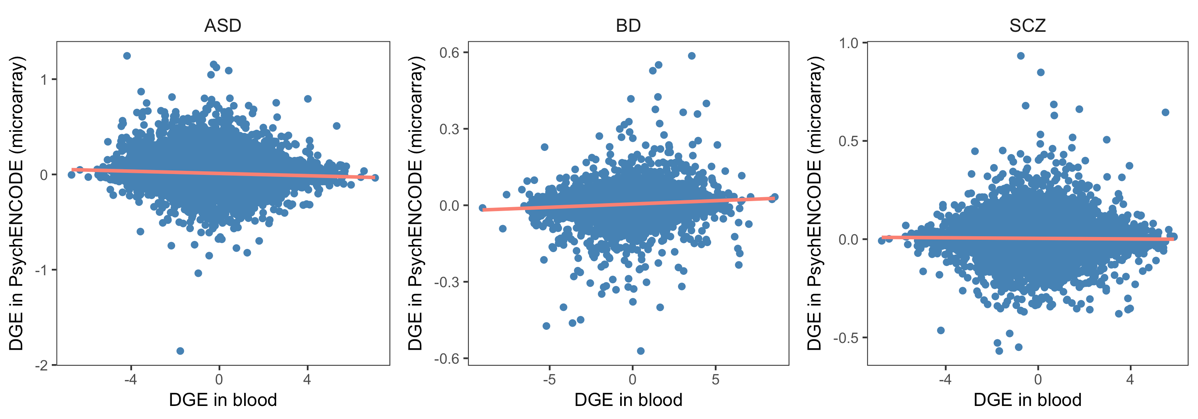 |
| **BrainGENIE DGE vs. PsychENCODE (microarray)**  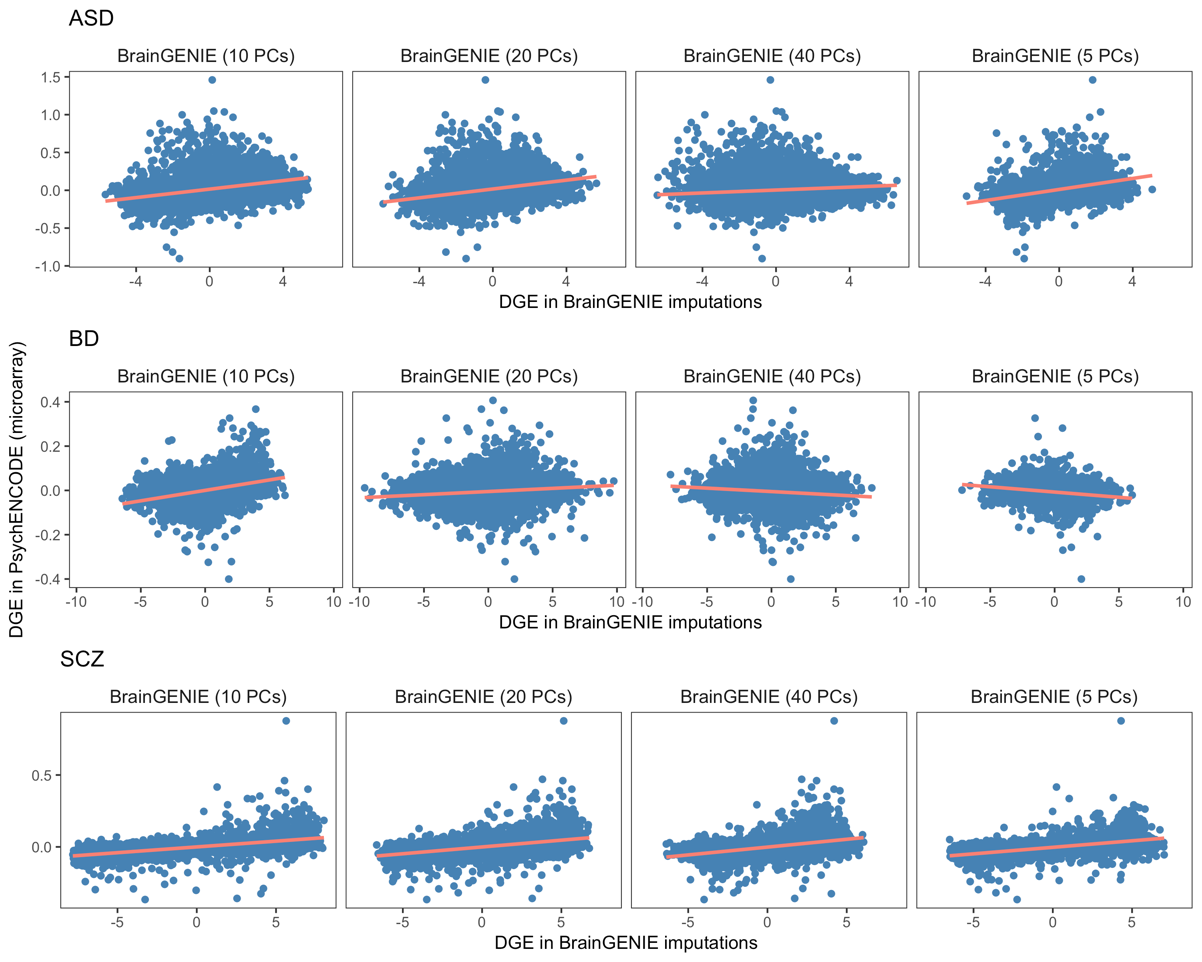 |
| **BrainGENIE DGE vs. PsychENCODE (RNA-seq)**  **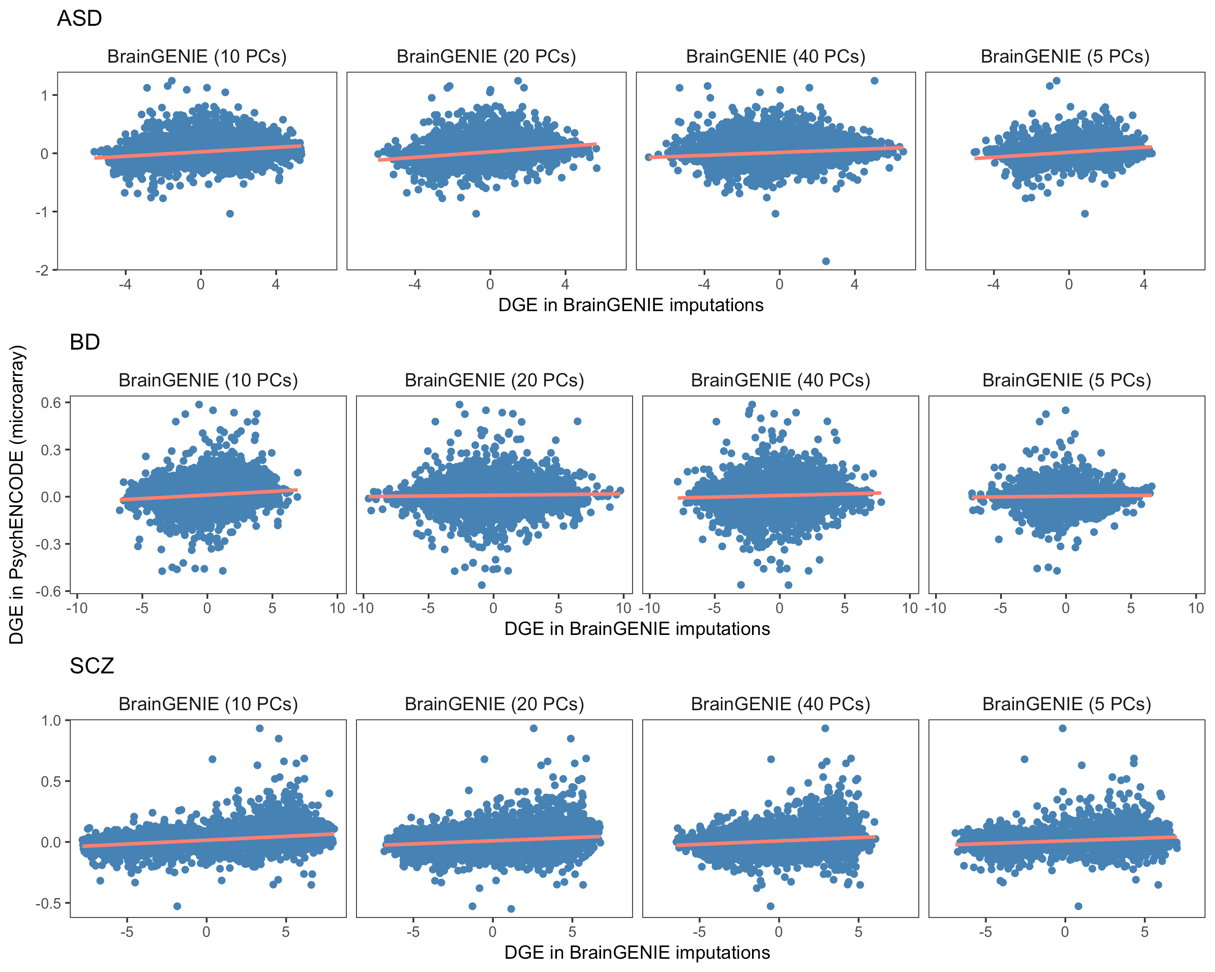** |
| **BrainGENIE DGE vs. CommonMind (RNA-seq)**  **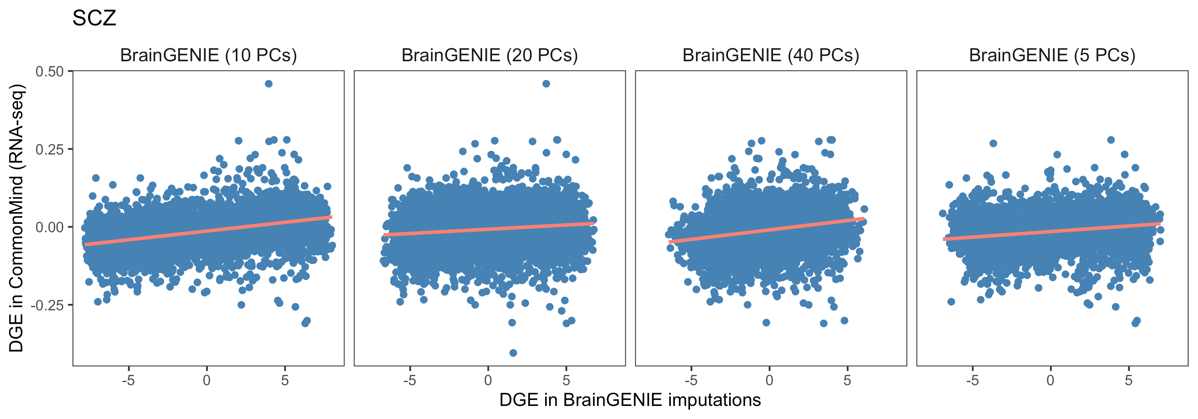** |
| **S-PrediXcan vs. PsychENCODE (microarray)**  **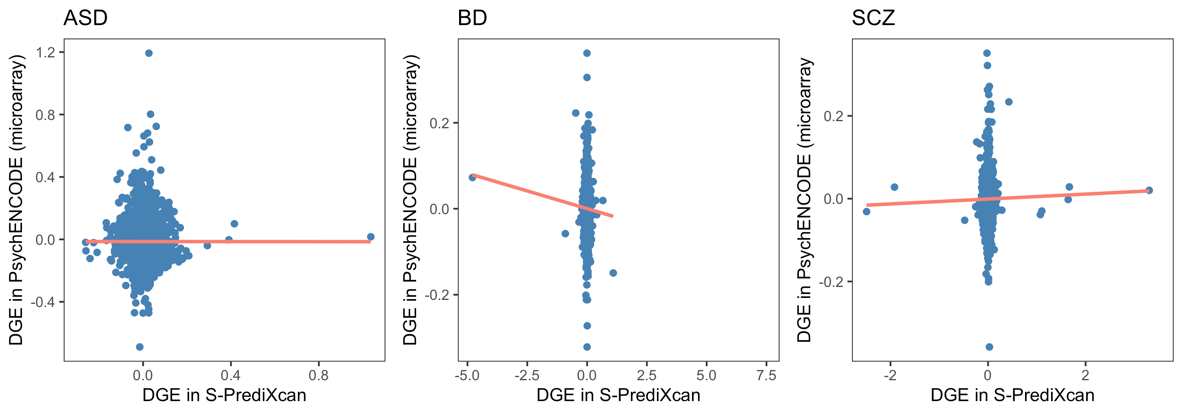** |
| **S-PrediXcan vs. PsychENCODE (RNA-seq)**  **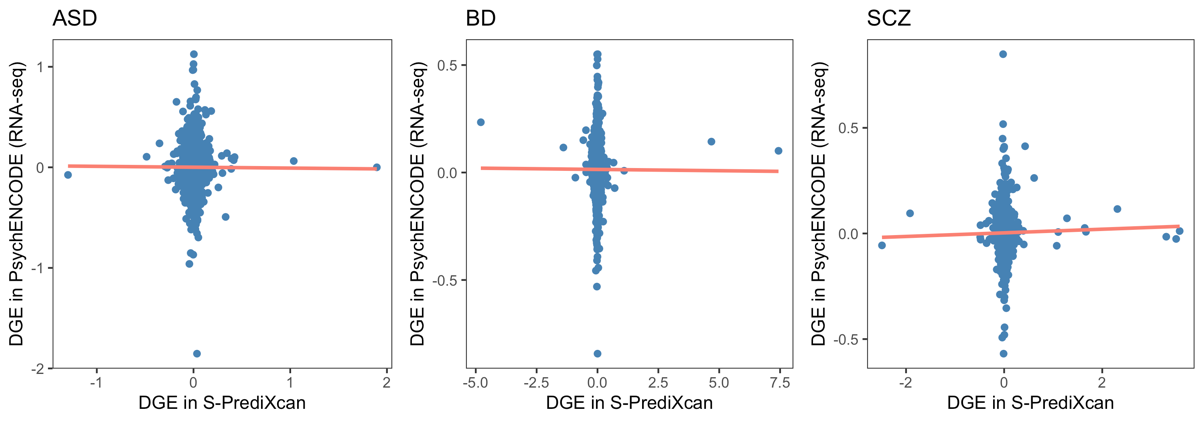** |
| **S-PrediXcan vs. CommonMind (RNA-seq)**  **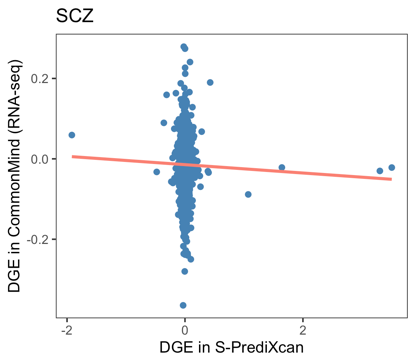** |

**Supplementary Figure 7.** Scatterplots depicting the relationship between differential gene expression effect sizes obtained in postmortem brain vs. blood, BrainGENIE imputations, and S-PrediXcan. These scatterplots are representative of the results that are presented in Figure 2 of our main text.

## **Literature Cited**

1. GTEx Consortium, Ardlie KG, Deluca DS, Segre A V., Sullivan TJ, Young TR, *et al.* (2015): The Genotype-Tissue Expression (GTEx) pilot analysis: Multitissue gene regulation in humans. *Science (80- )*. 348: 648–660.

2. Ritchie ME, Phipson B, Wu D, Hu Y, Law CW, Shi W, Smyth GK (2015): Limma powers differential expression analyses for RNA-sequencing and microarray studies. *Nucleic Acids Res*. . doi: 10.1093/nar/gkv007.

3. Newman AM, Liu CL, Green MR, Gentles AJ, Feng W, Xu Y, *et al.* (2015): Robust enumeration of cell subsets from tissue expression profiles. *Nat Methods*. . doi: 10.1038/nmeth.3337.

4. The GTEx Consortium (n.d.): Data downloaded from GTEx. . Retrieved from https://www.gtexportal.org/home/datasets.

5. Aguet F, Brown AA, Castel SE, Davis JR, He Y, Jo B, *et al.* (2017): Genetic effects on gene expression across human tissues. *Nature*. . doi: 10.1038/nature24277.

6. Hess JL, Tylee DS, Barve R, de Jong S, Ophoff RA, Kumarasinghe N, *et al.* (2016): Transcriptome-wide mega-analyses reveal joint dysregulation of immunologic genes and transcription regulators in brain and blood in schizophrenia. *Schizophr Res*. 176: 114–124.

7. Gautier L, Cope L, Bolstad BM, Irizarry RA (2004): affy--analysis of Affymetrix GeneChip data at the probe level. *Bioinformatics*. 20: 307–15.

8. Gardiner EJ, Cairns MJ, Liu B, Beveridge NJ, Carr V, Kelly B, *et al.* (2013): Gene expression analysis reveals schizophrenia-associated dysregulation of immune pathways in peripheral blood mononuclear cells. *J Psychiatr Res*. 47: 425–437.

9. Glatt SJ, Chandler SD, Bousman CA, Chana G, Lucero GR, Tatro E, *et al.* (2009): Alternatively Spliced Genes as Biomarkers for Schizophrenia, Bipolar Disorder and Psychosis: A Blood-Based Spliceome-Profiling Exploratory Study. *Curr Pharmacogenomics Person Med*. 7: 164–188.

10. Glatt SJ, Stone WS, Nossova N, Liew C-C, Seidman LJ, Tsuang MT (2011): Similarities and differences in peripheral blood gene-expression signatures of individuals with schizophrenia and their first-degree biological relatives. *Am J Med Genet B Neuropsychiatr Genet*. 156B: 869–87.

11. Kumarasinghe N, Beveridge NJ, Gardiner E, Scott RJ, Yasawardene S, Perera A, *et al.* (2013): Gene expression profiling in treatment-naive schizophrenia patients identifies abnormalities in biological pathways involving AKT1 that are corrected by antipsychotic medication. *Int J Neuropsychopharmacol*. 16: 1483–503.

12. de Jong S, Boks MPM, Fuller TTF, Strengman E, Janson E, de Kovel CGF, *et al.* (2012): A gene co-expression network in whole blood of Schizophrenia patients is independent of antipsychotic-use and enriched for brain-expressed genes. (M. Mazza, editor) *PLoS One*. 7: e39498.

13. Tsuang MT, Nossova N, Yager T, Tsuang M-M, Guo S-C, Shyu KG, *et al.* (2005): Assessing the validity of blood-based gene expression profiles for the classification of schizophrenia and bipolar disorder: a preliminary report. *Am J Med Genet B Neuropsychiatr Genet*. 133B: 1–5.

14. Beech RD, Lowthert L, Leffert JJ, Mason PN, Taylor MM, Umlauf S, *et al.* (2010): Increased peripheral blood expression of electron transport chain genes in bipolar depression. *Bipolar Disord*. 12: 813–24.

15. Bousman CA, Chana G, Glatt SJ, Chandler SD, Lucero GR, Tatro E, *et al.* (2010): Preliminary evidence of ubiquitin proteasome system dysregulation in schizophrenia and bipolar disorder: convergent pathway analysis findings from two independent samples. *Am J Med Genet B Neuropsychiatr Genet*. 153B: 494–502.

16. Clelland CL, Read LL, Panek LJ, Nadrich RH, Bancroft C, Clelland JD (2013): Utilization of never-medicated bipolar disorder patients towards development and validation of a peripheral biomarker profile. *PLoS One*. 8: e69082.

17. Padmos RC, Hillegers MHJ, Knijff EM, Vonk R, Bouvy A, Staal FJT, *et al.* (2008): A discriminating messenger RNA signature for bipolar disorder formed by an aberrant expression of inflammatory genes in monocytes. *Arch Gen Psychiatry*. 65: 395–407.

18. Savitz J, Frank MB, Victor T, Bebak M, Marino JH, Bellgowan PSF, *et al.* (2013): Inflammation and neurological disease-related genes are differentially expressed in depressed patients with mood disorders and correlate with morphometric and functional imaging abnormalities. *Brain Behav Immun*. 31: 161–71.

19. Krebs CE, Ori APS, Vreeker A, Wu T, Cantor RM, Boks MPM, *et al.* (2019): Whole blood transcriptome analysis in bipolar disorder reveals strong lithium effect. *Psychol Med*. . doi: 10.1017/S0033291719002745.

20. Witt SH, Juraeva D, Sticht C, Strohmaier J, Meier S, Treutlein J, *et al.* (2014): Investigation of manic and euthymic episodes identifies state- and trait-specific gene expression and STAB1 as a new candidate gene for bipolar disorder. *Transl Psychiatry*. 4: e426.

21. Hertz-Picciotto I, Croen LA, Hansen R, Jones CR, van de Water J, Pessah IN (2006): The CHARGE study: An epidemiologic investigation of genetic and environmental factors contributing to autism. *Environ Health Perspect*. . doi: 10.1289/ehp.8483.

22. Enstrom A, Krakowiak P, Onore C, Pessah IN, Hertz-Picciotto I, Hansen RL, *et al.* (2009): Increased IgG4 levels in children with autism disorder. *Brain Behav Immun*. . doi: 10.1016/j.bbi.2008.12.005.

23. Tian Y, Green PG, Stamova B, Hertz-Picciotto I, Pessah IN, Hansen R, *et al.* (2011): Correlations of gene expression with blood lead levels in children with autism compared to typically developing controls. *Neurotox Res*. . doi: 10.1007/s12640-009-9126-x.

24. Stamova B, Green PG, Tian Y, Hertz-Picciotto I, Pessah IN, Hansen R, *et al.* (2011): Correlations between gene expression and mercury levels in blood of boys with and without autism. *Neurotox Res*. . doi: 10.1007/s12640-009-9137-7.

25. Glatt SJ, Tsuang MT, Winn M, Chandler SD, Collins M, Lopez L, *et al.* (2012): Blood-based gene expression signatures of infants and toddlers with autism. *J Am Acad Child Adolesc Psychiatry*. 51: 934–44.e2.

26. Kong SW, Collins CD, Shimizu-Motohashi Y, Holm IA, Campbell MG, Lee IH, *et al.* (2012): Characteristics and Predictive Value of Blood Transcriptome Signature in Males with Autism Spectrum Disorders. *PLoS One*, 2012/12/12. 7: e49475.

27. Alter MD, Kharkar R, Ramsey KE, Craig DW, Melmed RD, Grebe TA, *et al.* (2011): Autism and increased paternal age related changes in global levels of gene expression regulation. *PLoS One*. . doi: 10.1371/journal.pone.0016715.

28. Kong SW, Shimizu-Motohashi Y, Campbell MG, Lee IH, Collins CD, Brewster SJ, *et al.* (2013): Peripheral blood gene expression signature differentiates children with autism from unaffected siblings. *Neurogenetics*. . doi: 10.1007/s10048-013-0363-z.

29. Lee PH, Anttila V, Won H, Feng YCA, Rosenthal J, Zhu Z, *et al.* (2019): Genomic Relationships, Novel Loci, and Pleiotropic Mechanisms across Eight Psychiatric Disorders. *Cell*. . doi: 10.1016/j.cell.2019.11.020.
